# Knowledge attribution, socioeconomic status, and education: new results using the Great British Class Survey

**DOI:** 10.1007/s11229-021-03131-6

**Published:** 2021-09-12

**Authors:** Boudewijn de Bruin

**Affiliations:** 1 Philosophy, University of Groningen, Oude Boteringestraat 52, 9712 GL Groningen, The Netherlands; 2 Economics, University of Groningen, Nettelbosje 2, 9747 AE Groningen, The Netherlands

**Keywords:** Gettier, Knowledge attribution/ascription, Socioeconomic status (SES), Great British Class Survey, Latent Class Analysis, Education

## Abstract

This paper presents new evidence on the impact of socioeconomic status (SES) and education on knowledge attribution. I examine a variety of cases, including vignettes where agents have been Gettiered, have false beliefs, and possess knowledge (according to orthodoxy). Early work investigated whether SES might be associated with knowledge attribution (Weinberg et al. in Philos Top 29(1–2):429–460, 2001; Seyedsayamdost in Episteme 12(1):95–116, 2014). But these studies used college education as a dummy variable for SES. I use the recently developed Great British Class Survey (Savage et al. in Sociology 47(2):219–250, 2013) to measure SES. The paper reports evidence against an association between SES and patterns of knowledge ascription, and reports mixed evidence about education effects.

**Supplementary Information:**

The online version contains supplementary material available at 10.1007/s11229-021-03131-6.

## Introduction

Do knowledge ascription judgments depend on socioeconomic status? Addressing this question (and the analogous one about culture) was among the key concerns driving groundbreaking work by Jonathan Weinberg, Shaun Nichols, and Stephen Stich (2001). These authors found that, indeed, Gettier judgments depend on socioeconomic status and culture, and they interpreted these findings as constituting a significant threat to epistemology as it was then conducted (or to “armchair” philosophy in general).^1^

But how stable are these findings really?

That laypeople judge differently than professional philosophers, in many Gettier cases, is by now beyond sensible doubt. Experimental philosophy has grown into a field of its own, and despite some objectors—embracing the *expertise defense*—many philosophers see the merit and relevance of such empirical research. Some replication studies about culture (Kim & Yuan, 2015; Machery et al., 2017a, 2017b; Nagel et al., 2013; Turri, 2013) and socioeconomic status (Seyedsayamdost, 2014b) suggest, however, that these results may not be too stable. As Machery and colleagues (2017) recently pointed out, these replication failures may have to do with (often) small sample sizes, not fully nationally representative samples, or with selection bias due to a sole focus on anglophone participants.^2^

In this paper, I raise another issue, which is specifically to do with socioeconomic status (SES). Empirical social scientists have typically measured SES through the following variables: highest achieved education level (of respondent and/or of parents of respondent); respondent’s annual household income; respondent’s assets/wealth (savings, property); or a combination of these variables. This is also the approach taken in experimental philosophy so far. Weinberg et al. (2001), for instance, defined low SES individuals as those not having attended college, and high SES as having one or more years of college.^3^

Such an approach has obvious disadvantages, though. With SES measured by educational achievements, the effects Weinberg et al. (2001) find may be driven by education rather than by SES. This may in fact weaken the threat posed to “armchair” philosophers, wedded as most of them are to the idea that what distinguishes laypeople and philosophers is expertise, gained through education. While one year of college education will not necessarily expose you to much philosophy, it might, the armchair philosopher could argue, have some effect, at least if we believe that college education boosts general intellectual capacities that have some role to play in knowledge ascription judgments. In other words, if it is educational differences rather than genuine socioeconomic differences that drive Weinberg et al.’s (2001) results, the threat these findings pose to armchair philosophy may be weaker.

In the discussion of the expertise defense, this point hasn’t been made yet, nor has the methodology of measuring SES through college education been questioned (Grundmann, 2010; Hofmann, 2010; Horvath, 2010; Kauppinen, 2007; Ludwig, 2010; Sorensen, 2014; Sosa, 2007). But if advocates of the expertise defense raised this issue, it would undeniably have some force.

Consequently, a preferable approach to detect potential SES effects uses a measure that is independent of education. Such a measure has recently been developed by a team of British sociologists led by Mike Savage et al. (2013): the Great British Class Survey. While this measure overlaps with earlier measures of SES in that it includes income and assets (a component of SES that Savage et al. (2013) call *economic capital*), it does *not* include education. Also, the Great British Class Survey includes two additional components, which are entirely missing from earlier measures: *social capital* (which captures the average status of one’s social contacts), and *cultural capital* (which represents the type of SES-relevant leisure activities one engages in). And also unlike most earlier work, Savage and colleagues deploy the sophisticated statistical machinery of Latent Class Analysis to derive, for every respondent, posterior probability estimates of socioeonomic class membership.

In this paper, I use the methodology of the Great British Class Survey to study the influence of SES on knowledge ascription. I report three studies. Study 1 involves a nationally representative sample of 2,824 UK nationals, recruited through the UK-based online portal Prolific. Study 2 involves a US sample of 1,059 respondents, sourced through Amazon Mechanical Turk. Study 3 is a UK sample of 820 respondents, also sourced through Prolific. Study 3 was a pilot study, conducted about a year before Studies 1 and 2. Following recent work by such scholars as Starmans and Friedman (2012), I expose participants to Gettier cases as well as to cases with false beliefs and to cases in which the consensus conception would attribute knowledge.

I find no evidence of statistically significant differences in knowledge ascription across different socioeconomic classes in any of these studies.

The data also allow me to probe whether there are any educational effects. Arguably, if we are interested in detecting potential educational effects on knowledge attribution judgments, then a more fine-grained measure than the dichotomous one used by Weinberg et al. (2001) (college education, yes or no?) should be preferable. So I used a measure specifically attuned to the UK educational system (in Studies 1 and 3) and the US (Study 2), and measure education in categories in line with national statistical conventions. In addition, I included a host of controls including gender, age, ethnicity, and so forth. With and without these controls in place, there is evidence of education effects in some studies, but not in all, and I do not therefore think we are in a position to claim with confidence that education is associated with knowledge attribution judgments. What is clear, however, is that with these controls in place SES still does not influence such judgments.

Despite the obvious relevance of these data to work on replication in experimental philosophy (e.g., the X-Phi Replicability Project (Cova et al., 2021)), the main concern in this paper is not replication. Rather, I aim to introduce to the experimental philosophy community a novel instrument to measure SES, as this allows us to separate SES effects and educational effects. I restrict myself here to knowledge attribution tasks, but I believe that the Great British Class Survey has interesting applications elsewhere in experimental philosophy as well.

I start this paper in Sect. 2 by providing some theoretical and empirical backing for why Weinberg et al. (2001) found the hypothesis about SES and Gettier judgments plausible. In Sect. 3 I introduce the Great British Class Survey. Sections 4–6 describe the experiments, and report the results. Section 7 concludes. The Appendix contains the texts of all vignettes as well as table work. Supplementary Materials contain information on the construction of the SES variables and other relevant matters, and will be made available online, together with the data.

## Socioeconomic status and cognition

One might wonder whether it still makes much sense—*even* in contemporary Britain—to speak about socioeconomic status, or social class. Aren’t we all middle class now, as former Labour Deputy Prime Minister John Prescott famously said? Or isn’t class merely a “communist concept” meant to set people against each other, as Margaret Thatcher thought? Perhaps yes: some class distinctions are certainly less important now than they were half a century ago. But 60 percent of the UK population still consider themselves “working class,” if asked which class (working, or middle class) they identify with most, a figure that has only changed a few percentage points since 2003 (Evans & Mellon, 2016). Moreover, income and wealth inequality have grown, with the mean annual household income of the top earning SES group in the Great British Class Survey at £89,000 ($145,000, at the time of conducting the survey), as compared to only £8,000 ($13,000) for the lowest earning group. But does this mean that members of different classes should be expected to make different judgments about Gettier cases? Weinberg et al. (2001) referred to work by Jonathan Haidt and colleagues (1993) to answer this question affirmatively. Haidt and colleagues showed that moral intuitions witness considerable differences between high SES and low SES individuals.^4^ They presented individuals with vignettes of “victimless yet offensive” actions (such as not keeping a promise after the promisee dies, or eating the meat of the family dog after it has died), and found that high SES individuals judge these vignettes through the lens of social convention or personal preference, whereas low SES individuals assume a moralizing attitude towards them.

Weinberg et al. (2001) used Haidt et al. (1993) as their reference point. But what does the more recent social science literature say about SES?^5^ Evidence is mixed, to say the least, as the replicability of several key results is intensely debated. To give one example, an oft-cited paper argued that higher SES individuals engage in unethical behavior more often than lower SES individuals (Piff et al., 2012), but various replication attempts have failed (Balakrishnan et al., 2017; Clerke et al., 2018). This is one reason why we should be very careful when we wish to build our experimental philosophy hypotheses on prior work in the social sciences. Perhaps just as important for our purposes is the way these studies measure SES. As I said in the introduction, the traditional approach is to use proxies for SES such as income, wealth, or educational achievement. As a result, most of the studies that might be potentially relevant to developing hypotheses about the association between SES and Gettier judgments would, strictly speaking, be relevant only to a hypothesis on the association between such judgments and the specific economic or educational variables (which are used as proxies for SES). That is a second reason why I am reluctant to turn to published research to motivate the project. I believe, however, that the use of a new instrument to measure SES independently of education is sufficiently interesting in itself to merit further attention from philosophers.

## The Great British Class Survey

The Great British Class Survey was launched in 2011 in order to overcome a number of drawbacks of earlier measures of social class that had been articulated in the scholarly literature (data collection 2011–2013). I briefly summarize earlier measures and criticism. In the 1970s, John Goldthorpe of Nuffield College, Oxford, had developed a highly influential class schema known as the Nuffield class schema, or Erikson–Goldthorpe–Portocarero (EGP) model. Seven classes were distinguished based on a person’s employment position. Employees and employers were set apart, and amongst employees, those on labor contracts (routine, semi-routine, and technical employees) were distinguished from employees with service relationships with their employers.^6^

While the EGP model was very influential in academia and policy circles, it was criticized on many counts. The focus on occupational status was claimed to obscure class distinctions pertaining to social activities and cultural consumption (Devine, 1998). It was tailored to be useful in fairly small sample sizes, with the result of making it improbable to discover very small “microclasses” (e.g. a “one percent” wealthy elite) (Savage & Williams, 2008). Economists, moreover, criticized the model on the ground that a focus on occupational groups led to a skewed view of class inequality in an economy where income and wealth differences within occupations increase (Jenkins, 2011). Feminist scholars pointed out the potentially stigmatizing effect the EGP model (Skeggs, 1997). And scholars such as Oesch (2006) argued that the EGP approach is of questionable validity in comparative studies of SES in Europe.

The Great British Class Survey (GBCS), developed by Mike Savage and colleagues (2013), is an attempt to allay these criticisms. The core innovation is to include next to economic variables of income and wealth (that is, *economic capital*), two further clusters of variables. One captures *social capital*, and is measured by the number of social contacts (from a given list), and the weighted score of these contacts (where scores reflect the “social standing” of the contacts). The other cluster of variables captures *cultural capital*, and is measured by the type of activities one engages in (from a list of activities that distinguish emerging culture from high culture). I briefly discuss these variables here. More details follow in the methods sections of the three studies. Supplementary Materials contain further details on variable construction.

*Economic capital* Economic capital was measured by asking respondents to report their total annual household income after tax (*Inc*), the value of the house they own (coded 0 if rented), and the current value of their savings aside from the house, all presented as categorical questions. Midpoints of the categories were used for variable construction, and savings and house value were taken together (*Assets*).

*Social capital* Social capital was measured using the position generator approach due to Lin (2001). Respondents were asked to report whether they socially know people from a list of given occupations (secretary, nurse, teacher, cleaner, university lecturer, artist, electrician, office manager, solicitor, farm worker, chief executive, software designer, call center worker, postal worker, scientist, lorry driver, accountant, shop assistant). The variable *Occstatus* is the weighted sum of the number of social contacts they check, divided by the total number of all occupations in the list, with weights derived from the Cambridge Social Interaction and Stratification Scale. The variable *Occnumber* is the mere number of occupations respondents report to know socially.

*Cultural capital* Cultural capital was measured by two variables. Savage and colleagues (2013) adopted an inductive approach. They chose a list of activities that they expected to be either characteristic of high or of emerging cultural capital (e.g., opera, gardening, hip-hop/rap). Participants were asked to tell how much they engaged in these activities, and on the basis of their answers, two subsets of these activities were chosen, using multiple correspondence analysis. This is a statistical technique that reduces a variety of measures (here more than thirty items about activities) to dimensional variables (here one for high and one for emerging cultural capital), in a way that is not unlike factor analysis. Some of the original activities turned out not to be sufficiently strongly associated with either high or emerging culture, and so they were omitted (e.g., gardening). So in the end, two dimensional variables were constructed. The variable *Actemer* counts the number of activities that are characteristic of emerging cultural capital (computer games, social media, playing sports, watching sports, socializing with friends, going to the gym, going to gigs, hip-hop/rap, surfing the internet, rock/indie), while *Acthigh* counts the number of high cultural capital activities (preference for French food, arts and crafts, museums/galleries, stately homes, theater, opera, classical music, modern jazz, dance/ballet).

Savage and colleagues (2013) use Latent Class Analysis (LCA) to determine SES classes on the basis of information about these six variables (income, assets, number of contacts, mean social score of contacts, emerging culture score, high culture score), such that every respondent can be assigned to one particular class. Very roughly, the starting point is information about six variables $y_{{i1}} , \ldots ,y_{{i6}}$ for every $i$. LCA then estimates a particular model that ultimately allows us to determine, for each respondent $i$ and each SES group $x$, the (posterior) probability that $i$ belongs to $x$. This procedure is iterated for one, two, three classes, and so on, and then a model is chosen satisfying particular *information criteria*.^7^

LCA is related to such multivariate techniques as principal component analysis and factor analysis, but as Savage and colleagues argue (2013), it is the preferred methodology for present purposes because it does not presuppose, as principal component analysis and factor analysis do, that the variables are continuous and normally distributed. Since it is plausible to assume that SES is a categorical construct (or at least that we want to leave that option open), LCA is the preferred technique.^8^ However, it should be pointed out that researchers may introduce non-statistical pragmatic or theoretical reasons to choose models that are not optimal from the AIC or BIC point of view. For instance, Savage et al. (2013) use seven instead of eight classes for their GBCS, Albert et al. (2018) use eight instead of twelve classes in their SES measure for Hungary, and Sheppard and Biddle (2017), in a study in which they use the GBCS methodology to get an SES measure for Australia, use the number of classes determined by AIC, but discard BIC, for theoretical, sociological reasons rather than statistical considerations. I should also acknowledge a strand of literature that criticizes the use of LCA in sociology (Mills 2014). As for any statistical technique, the results of conducting LCA should be used and interpreted with care.

Details about LCA and the methodology of the GBCS are provided by Vermunt et al. (2002) and Savage et al. (2013), as well as in the Supplementary Materials. What is important here is that Savage and colleagues found evidence for the existence of seven classes, which I describe here below.

*Elite* Members of the Elite score highest on income and assets, have large numbers of social contacts, and score highest on high culture. Chief Executive Officers and other top managers are over-represented in this class, as are dentists and barristers. It includes a large share of graduates from elite universities such as Cambridge and LSE.

*Established Middle Class* Members of the Established Middle Class have an average annual household income about half of that of members of the Elite, but are still very well off. It is a large class, about a quarter of the UK population, with the highest number of social contacts. Culturally, they are omnivores. While a fairly large number of people in this class are university graduates, there is a much greater variety of occupations than in the Elite. Over- represented occupations include occupational therapists, midwives, police officers, among others.

*Technical Middle Class* The discovery of this small class, which comprises only 6% of the UK population, is one of the key new findings of Savage et al. (2013). (See above why earlier measures were unable to detect it.) This class is about as wealthy as the Established Middle Class, but has very distinct social and cultural capital. It includes graduates in science and technology, and occupations such as aircraft pilots, medical radiographers, or pharmacists. It has the lowest number of social contacts of all classes (but of highest status). Savage and colleagues hypothesize that social interaction is restricted to members of the same class here. Culturally, the Technical Middle Class seems to be rather disengaged, and is characterized by “relative social isolation” and “cultural apathy” (Savage et al., 2013, 237).

*New Affluent Workers* Members of the class of New Affluent Workers score high on emerging, and low on high culture. They are economically less well off than the previous classes, but certainly secure. They often come from less well off families. Few of them are university graduates. Characteristic of the New Affluent Workers is that they have accomplished economic security through non-conventional routes.

*Traditional Working Class* This class is not very well off, even though most members own their own house. Members score low both on high and emerging culture, and their social contacts are limited in terms of status, but high in number. Over-represented occupations include lorry drivers, cleaners, and other occupations traditionally associated with “working class.”

*Emergent Service Workers* This class has a mean emerging culture score higher than any other class. Economic capital is low. It includes a high proportion of ethnic minorities, and is socially active. With the Traditional Working Class they share relatively modest backgrounds, but they differ greatly in terms of cultural engagement. Occupations include work in restaurants and bars, customer services, or call centers. Interestingly, this class includes an over-representation of graduates in arts and humanities.

*Precariat* The Precariat is economically poorest, with a small range of social contacts, and exceedingly low scores on both high and emerging culture. It is the “most deprived” class (Savage et al., 2013, 243), with many of its members in old industrial areas, including many unemployed people, cleaners, carpenters, and cashiers.

## Study 1

### Method

I conducted an a priori power analysis using the G*Power 3.1 software package (Faul et al., 2007). I assumed a moderate effect size (*w* = .3) and set *α* = .05 and *β* = .95, in line with current practices in the replication literature in experimental philosophy (Cova et al., 2021), as well as with Cohen’s (1988) and Sedlmeier and Gigerenzer’s (1989) recommendations for testing null hypotheses. This led to the number of 232 respondents per vignette (chi-square test, with df = 6, for the seven-class GBCS model), or 1,624 respondents in total.^9^ I acknowledge that the assumption of a moderate effect size can be criticized. Assuming an effect size of *w* = .2 or *w* = .1, respectively, would, however, have required a total of 3,654 or 14,602 respondents, respectively, which far exceeds the available research budget. However, the sample size of 1,624 that I aimed for is an upper limit: it is based on the seven-class SES measure, but I also planned to use less discriminatory, dichotomous variables for SES (measuring low SES and high SES, and also membership of each of the individual seven classes). With the assumption of a small effect size (*w* = .1), *α* = .05, and *β* = .95, we would need 1,300 observations, with such dichotomous variables.

The pilot study (Study 3) had given rise to some questions about the quality of observations gathered through the Prolific platform as no comprehension checks were used.^10^ To err on the conservative side, I estimated that in order to maintain sufficient power after discarding respondents failing these checks, we would need a sample 1.5 times larger than 1,624 respondents, that is, 2,436 respondents. As Prolific only allows nationally representative samples to a maximum of 1,500 respondents, I had to source twice. In the second stage, conducted the same day, I accidentally sampled more than the required amply 900, arriving at an ultimate total sample size of 2,824.

*Participants* Two thousand eight hundred twenty-four respondents (1,418 female, mean age 41 years, UK residents) were recruited, using Prolific, an alternative to Amazon Mechanical Turk (MTurk). Due to its large presence in the UK and its ability to generate samples approximating UK national representativeness, Prolific was the preferred choice given my intention to use the Great British Class Survey as a measure of SES. The questionnaire was distributed through Qualtrics. I used exclusion criteria as in Starmans and Friedman (2012), and thus removed unfinished tasks (90), duplicate IP addresses (102), observations with completion times less than 60 or more than 1,000 seconds (207), observations of respondents who indicated that they had participated in a similar experiment before (500), and observations with one or more failed comprehension questions (of a total of four) (576). So I removed a total of 1,160 observations, and obtained a sample of 1,664 participants (878 female, mean age 42 years). Participants were paid the recommended Prolific rate of £0.50 ($0.65) per five minutes of their time.

*Materials and procedure* The experiment involved seven vignettes, all drawn from the literature. They included three Gettier cases, a case where there is no knowledge because the belief is not true, and three cases where there was knowledge: Watch (Gettier), Banknote (false belief), and Book (knowledge) (Experiment 1A (Starmans & Friedman, 2012), with “banknote” instead of “dollar bill” to adjust to the UK context), Car (Gettier) (Weinberg et al., 2001), Match (knowledge) (Beebe & Shea, 2013), Trip (Gettier) (Nagel et al., 2013), and Politician (knowledge) (Beebe & Shea, 2013), some with histories in the theoretical literature.^11^ The Appendix contains the texts of the vignettes. Questions were of the form: “Which of the following is true?” with possible answers in random order, for instance: “Bob really knows that Jill drives an American car,” and “Bob only thinks that Jill drives an American car.” Standard demographic questions were asked (age, gender, etc.). Questions from the GBCS were included. Supplementary Materials give full details. To measure economic capital, respondents were asked to select the best fitting categorical income, property, and savings range (e.g., “What is your annual household income after taxes?” Possible answers: “Under £5,000,” “£5,000 to £10,000,” etc.). Social capital was measured by asking participants questions about their social network. Participants were asked: “Think about your family, friends and acquaintances. What do they do? Here is a list of occupations. For each one, select it if you know someone socially who does that.” This was followed by a list of occupations (secretary, travel agent, call center worker, etc.). Supplementary Materials contain the list of all occupations. Questions about cultural activities, preferences for music, and restaurant preferences captured participants’ cultural capital. Participants were told: “You will now be asked to consider a number of activities, and asked to indicate how much you participate (never, rarely, sometimes, often).” This was followed by a list (watching TV, playing computer games, reading books, etc.). Similar questions were asked about music preference (rock/indie, classical/opera, etc.) and restaurant preferences (café or teashop, pizza restaurant, etc.).

Posterior probabilities of SES group membership were derived by combining my data with the data from Savage and colleagues (2013). Supplementary Materials contain details of this procedure.

Education was measured as follows. In line with UK statistical conventions, seven educational classes were distinguished (1 = “GCSE/O level below grade C, qualifications at level 1 or below,” 2 = “GCSE/O level grade A*–C, vocational level 2, or equivalent,” 3 = “A level, vocational level 3, or equivalent,” 4 = “Higher education below degree level,” 5 = “Bachelor’s degree or equivalent level,” 6 = “Master’s degree or equivalent level,” 7 = “Doctoral degree or equivalent level”).

Besides these questions, I included in the survey also the original comprehension checks from Starmans and Friedman’s (2012) experiment as well as their questions about prior exposure to philosophy and similar experiments. For the vignettes drawn from other sources, I designed maximally analogous comprehension checks. I also introduced a very rigorous attention check, frequently used in the economics lab of the University of Groningen, which to the careless reader seems to ask for their astrological sign, but in reality asks them to state their favorite sport. The Supplementary Materials contain details of the comprehension questions and attention check. The questionnaire ended with three unrelated items about the Covid-19 pandemic and political identity, for use in another study.

### Results

#### Replication of published studies

The most common outcome variable takes a respondent’s answer to the knowledge attribution question (knowledge/belief) as a dichotomous variable. In addition to this, Starmans and Friedman (2012) calculate a respondent’s Weighted Knowledge Ascription (WKA), where knowledge ascription is weighted by the confidence the respondent has in the answer, measured on a ten-point Likert scale. Coding knowledge ascription as 1 and belief ascription as −1, WKA is equal to the confidence level when knowledge is attributed, and minus (that is, −1 ×) the rescaled confidence level if the respondent does not attribute knowledge. So, for instance, if a respondent reports a confidence level of 7 and ascribes belief, their WKA will be −7. WKA is, then, a 20-point scale, ranging from −10 to 10, excluding 0.

In contrast to the dichotomous measure, WKA may not have an entirely straightforward interpretation. In what sense, for instance, would two respondents who with confidence levels of 2 disagree in their knowledge ascription (so they have WKAs of 2 and −2, respectively) be closer to each other than two respondents who ascribe knowledge but differ in confidence (say, with WKAs of 10 and 5, respectively)? This is not to do with the use of a Likert scale for confidence, or with the specific range used for the Likert scale. Rather my reservations concern the fact that WKA assigns a “weight” to a respondent’s judgment, namely, the confidence they have in their judgment. Clearly, assigning weights is not always unproblematic. The concept of expected utility, where one’s utility is weighted by one’s probabilistic expectations, comes to mind, but in that case, the Von Neumann–Morgenstern axioms (and their representation theorem) help us to get a plausible interpretation for the weighted construct. It is not clear upfront that something along those lines could be done to back WKA. That is why I report WKA in so far as it allows me to compare my results with those of Starmans and Friedman (2012). But the focus in my analysis lies on the dichotomous outcome variable.

**Fig. 1:**
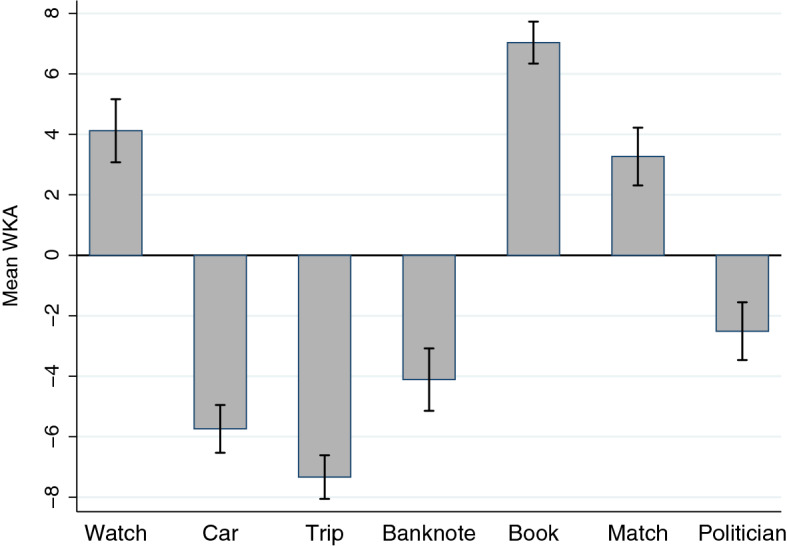
Weighted Knowledge Ascription per Vignette (Study 1) *n* = 1,664. The figure displays the mean weighted knowledge ascription (WKA) per vignette, which was significantly different from chance in all vignettes (see main text). Error bars indicate 95% confidence intervals. Watch, Car, and Trip are Gettier cases. Banknote is a false belief case. Book, Match, and Politician are knowledge cases

WKA was significantly different from chance in all cases: Watch (a Gettier case, *M* = 4.12, *SD* = 7.47, *t*(198) = 7.78, *p* < .001, all two-sided), Banknote (a false belief case, *M* =  − 4.11, *SD* = 7.82, *t*(223) =  − 7.86, *p* < .001), Book (a knowledge case, *M* = 7.03, *SD* = 6.04, *t*(291) = 19.92, *p* < .001), Car (Gettier, *M* =  − 5.74, *SD* = 6.50, *t*(262) =  − 14.33, *p* < .001), Match (knowledge, *M* = 3.27, *SD* = 6.72, *t*(262) = 6.72 *p* < .001), Trip (Gettier, *M* =  − 7.34, *SD* = 4.72, *t*(166) =  − 20.09, *p* < .001), and Politician (knowledge, *M* =  − 2.51, *SD* = 7.77, *t*(255) =  − 5.17, *p* < .001). Figure 1 displays WKA per vignette.

**Fig. 2:**
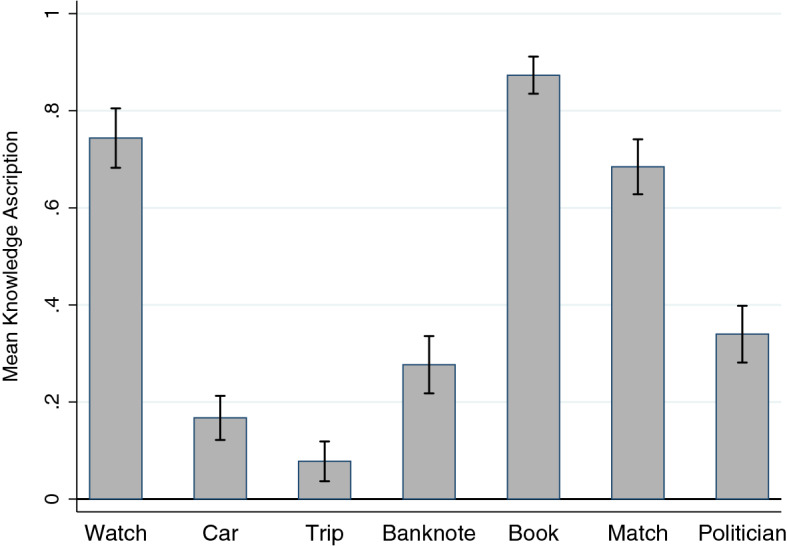
Knowledge Ascription per Vignette (Study 1) *n* = 1,664. The figure displays the mean knowledge ascription per vignette, which was significantly different from chance in all vignettes (see main text). Error bars indicate 95% confidence intervals. Watch, Car, and Trip are Gettier cases. Banknote is a false belief case. Book, Match, and Politician are knowledge cases

Considering the dichotomous knowledge attribution questions (knowledge/belief), I found knowledge ascription rates significantly different from chance in all cases (binomial, *p* < .001, all two-sided): Watch (Gettier, 74%), Banknote (false belief, 28%), Book (knowledge, 87%), Car (Gettier, 17%), Match (knowledge, 68%), Trip (Gettier, 8%), and Politician (knowledge, 34%). Figure 2 displays the mean knowledge ascription rate in each of the seven vignettes.

I replicated Watch (a Gettier case, Starmans and Friedman (2012), *Z* = .29, *p* = .775, two-sided difference in proportion test/z test, 72% of 47 respondents in the original study, 74% of 199 respondents here), Book (a knowledge case, Starmans and Friedman (2012), *Z* = .18, *p* = .86, 88% of 51 respondents in the original study, 87% of 292 respondents here), Car (Gettier, Kim and Yuan (2015), *Z* = .55, *p* = .583, 14% of 58 respondents, 17% of 263 respondents here), and Match (knowledge, Beebe and Shea (2013), *Z* = 1.25, *p* = .213, 78% of 41 respondents, 68% of 263 respondents here). I did not replicate Banknote (false belief, Starmans and Friedman (2012), *Z* = 2.40, *p* < .05, 11% of 46 respondents, 28% of 224 respondents here), Trip (Gettier, Machery et al., (2017a, 2017b), *Z* = 5.74, *p* < .001, 39% of 64 respondents, 8% of 167 respondents here), and Politician (knowledge, Beebe and Shea (2013), *Z* = 4.42, *p* < .001, 65% of 60 respondents, 34% of 256 respondents here).

To determine the robustness of the measure, I compared various samples, ranging from the entire dataset (2,824 observations) to the most stringent exclusion criteria, where I discarded unfinished observations, duplicate IP addresses, outlier completion times (less than 60 or more than 1,000 seconds), failed comprehension checks, having taken one or more philosophy courses, having participated in a similar experiment, or failed the stringent astrology/sports attention check (996 observations). For none of these samples do knowledge ascription rates change substantially, so the replication pattern remains. The Supplementary Materials contain information on the exact percentages and about the differences between my experiments and those reported in the published literature that might account for the differences.

#### Estimating SES effects

*Pairwise comparisons* To begin with, I conducted an array of simple pairwise comparisons of knowledge attribution judgments across SES classes. I here used various subsamples of observations: pairwise comparisons for each vignette individually, and pairwise comparisons for the following groups of vignettes: all vignettes together, the Gettier vignettes together (Watch, Car, Trip), the knowledge vignettes together (Book, Match, Politician), the replicated vignettes together (Watch, Book, Car, Match), and the non-replicated vignettes together (Banknote, Trip, Politician).

**Fig. 3:**
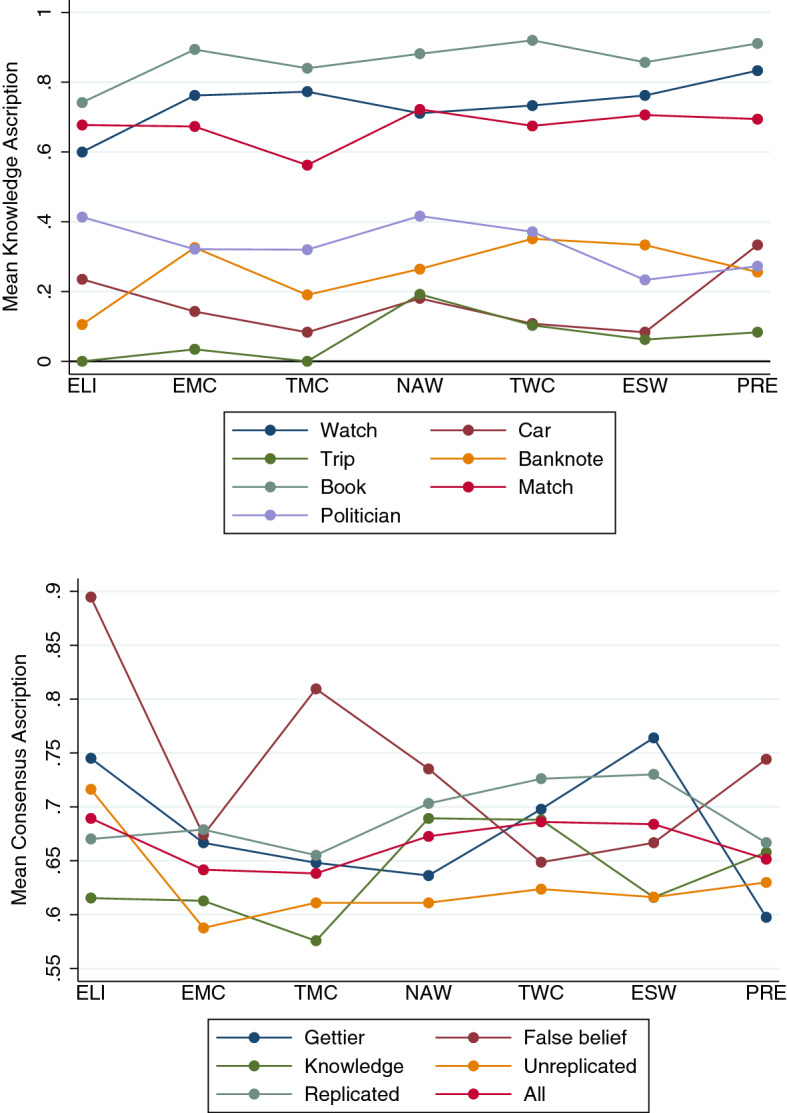
Knowledge Ascription and SES per Vignette, and per Group of Vignettes (Study 1) The graphs in the upper panel plot the mean knowledge ascription for each of the seven SES groups, per vignette (*n* = 1,664). Watch, Car, and Trip are Gettier cases. Banknote is a false belief case. Book, Match, and Politician are knowledge cases. The graphs in the lower panel plot the mean consensus ascription for each of the seven SES groups, for various groups of vignettes. Consensus ascription is 1 if the knowledge/belief question was answered in line with epistemological consensus, and 0 otherwise. Scale is from .55 to .90. Gettier (*n* = 629) includes Watch, Car, and Trip. False belief (*n* = 224) includes Banknote. Knowledge (*n* = 811) includes Book, Match, and Politician. Unreplicated (*n* = 647) includes Banknote, Trip, and Politician. Replicated (*n* = 1,017) includes Watch, Book, Car, and Match. All (*n* = 1,664) includes all seven vignettes. ELI = “Elite,” EMC = “Established Middle Class,” TMC = “Technical Middle Class,” NAW = “New Affluent Workers,” TWC = “Traditional Working Class,” ESW = “Emergent Service Workers,” and PRE = “Precariat.” The order of the seven groups follows Savage et al. (2013)

I used two outcome variables: *knowledge ascription* (1 if the respondent ascribes knowledge, 0 otherwise), and *consensus ascription* (1 if the respondent’s judgment aligns with epistemological consensus or orthodoxy, 0 otherwise). I consider knowledge ascription for the individual vignettes, and consensus ascription for the grouped vignettes. The reason is the following. For one given vignette, the consensus ascription pattern across SES classes either coincides with or is the mirror image of the knowledge ascription pattern. Yet, for any group of vignettes containing two or more vignettes with opposite consensus ascription (e.g., Watch in which “belief” is the consensus answer, and Book in which “knowledge” is the consensus answer), the knowledge ascription pattern and the consensus ascription pattern may look different. In this paper, I am ultimately interested in whether differences in SES class membership correlate with differences in consensus ascription rather than knowledge ascription per se, and that is why for the grouped vignettes consensus ascription was used.^12^ Among these more than two hundred pairwise comparisons (with Bonferroni correction), none comes out statistically significant, giving some initial evidence that SES is uncorrelated with knowledge attribution judgments.^13^ The two graphs in Fig. 3 plot knowledge ascription (upper panel) and consensus ascription (lower panel) against SES, for each of the pairwise comparisons conducted.

*Regressions* I performed an array of logistic regressions on knowledge attribution with a variety of predictors capturing SES. I considered the subsamples also studied in the pairwise comparison (each vignette individually, all vignettes together, Gettier vignettes, etc.).

I first used the SES classes as dichotomous variables *Elite* (1 = “Elite,” 0 otherwise), *EMC* (1 = “Established Middle Class,” 0 otherwise), and so on for all remaining SES classes (seven separate regressions, here and elsewhere). No statistically significant relation was found between these variables and knowledge ascription, on the sample of all vignettes taken together: *Elite*, *χ*^2^(1) = .45, *p* = .502; *EMC*, *χ*^2^(1) = 1.03, *p* = .308; *TMC*, *χ*^2^(1) = .52, *p* = .471; *NAW*, *χ*^2^(1) = .09, *p* = .767; *TWC*, *χ*^2^(1) = .56, *p* = .453; *ESW*, *χ*^2^(1) = .36, *p* = 548; *Pre*, *χ*^2^(1) = .26, *p* = .611 (all Nagelkerke *R*^2^ = .000 except for *EMC*, Nagelkerke *R*^2^ = .001).

Next I estimated models with two further dichotomous variables distinguishing between high SES and low SES individuals: *HighSES* (1 for Elite or Established Middle Class, 0 otherwise), and *LowSES* (1 for Traditional Working Class or Precariat, 0 otherwise). These models, too, were not statistically significant: *HighSES*, *χ*^2^(1) = .20, *p* = .652; *LowSES*, *χ*^2^(1) = .04, *p* = .844, both Nagelkerke *R*^2^ = .000. Power as computed using G*Power was high (1.0), assuming a moderate effect size (*w* = .3) and *α* = .05 (Faul et al., 2007). Total sample size here is 1,664 observations, as all vignettes were included.^14^

Finally, I studied a model with an ordinal measure of SES, *SES*. This predictor takes into account the order of the seven classes as suggested by Savage et al. (2013) (1 = “Elite,” 2 = “Established Middle Class,” 3 = “Technical Middle Class,” 4 = “New Affluent Workers,” 5 = “Traditional Working Class,” 6 = “Emerging Service Workers,” and 7 = “Precariat”). No statistically significant results were found (*p* = .778). Taking *SES* as a categorical variable does not change the findings (*χ*^2^(6) = 2.80, *p* = .834, Nagelkerke *R*^2^ = .002). Assuming a moderate effect size (*w* = .3) and *α* = .05, power was high (1.0).^15^

**Fig. 4:**
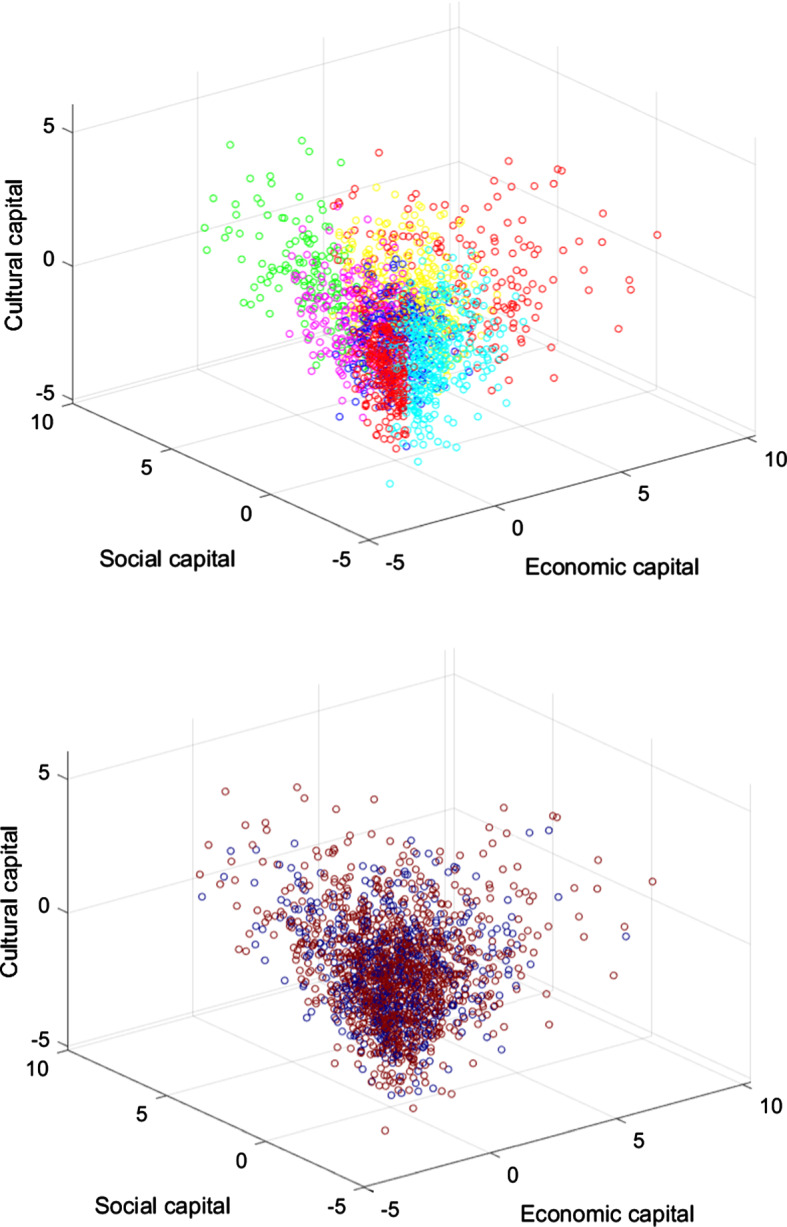
Economic, Social, and Cultural Capital and SES Group Membership, and Consensus Ascription (Study 1) *n* = 1,664. The graphs plot each subject in three-dimensional space. Economic capital is measured by the sum of income and assets. Social capital is the sum of the mean status of one’s social contacts and the number of one’s social contacts. Cultural capital is the sum of the high and emerging culture scores. For the six underlying variables standardized versions are used. In the upper panel, the color of each subject represents the SES group to which they are assigned using Latent Class Analysis as per Savage et al. (2013). Orange = “Elite,” yellow = “Established Middle Class,” green = “Technical Middle Class,” blue = “New Affluent Workers,” indigo = “Traditional Working Class,” violet = “Emergent Service Workers,” and red = “Precariat.” In the lower panel, the color of each subject represents whether they answered the knowledge/belief question according to epistemological consensus (red) or not (blue). No noise is added to observations to duplicate coinciding observations

Table 1 in the Appendix contains relevant regression results, and Fig. 4 goes some way to illustrating these results. The upper panel plots all observations in three-dimensional space (representing the three dimensions of SES as per Savage et al. (2013): economic, social, and cultural capital), with colors for posterior SES group membership as determined by Latent Class Analysis. The lower panel plots the same observations, with colors for consensus ascription. In the upper panel, the seven SES classes occupy fairly distinct regions in the graph, representing the way LCA has clustered them. If SES and consensus ascription were correlated, one should have expected to be able to trace these clusters back in the lower panel to some extent: some SES groups should tend to follow consensus, and others not. What we see, however, is more random than that.

These findings remain unchanged if we run the regressions over individual vignettes, or over specific groups of vignettes. For Gettier cases, regressing with the *SES* variable (taken as ordinal), for instance, yields *p* = .673. Of the amply one hundred regressions, only three show statistically significant results, namely, in three regressions on individual vignettes, using *Elite* in Book (a knowledge case, *χ*^2^(1) = 4.48, *p* < .05, Nagelkerke *R*^2^ = .029, correctly classifying 87.3% of the data points, 292 observations, decreasing knowledge ascription by 15 percentage points), *NAW* in Trip (a Gettier case, *χ*^2^(1) = 4.44, *p* < .05, Nagelkerke *R*^2^ = .062, correctly classifying 92.2% of the data points, 167 observations, increasing knowledge ascription by 14 percentage points), and *Pre* in Car (Gettier, *χ*^2^(1) = 7.77, *p* < .01, Nagelkerke *R*^2^ = .049, correctly classifying 83.3% of the data points, 263 observations, increasing knowledge ascription by 19 percentage points). Assuming moderate effect size (*w* = .3) and *α* = .05, the power of these three tests was 1.0, 0.97, and 1.0, respectively.

These three results are, however, not very robust. The robustness check here is to see if a slightly more encompassing indicator of SES preserves the three results. First, *Pre* in Car. If we construct a new SES indicator variable for the two lowest SES classes (so 1 = “Precariat or Emerging Service Workers,” 0 otherwise), which is identical to *LowSES*, then the model is no longer statistically significant (*χ*^2^(1) = 2.33, *p* = .127, Nagelkerke *R*^2^ = .015, 263 observations). Then, *Elite* in Book. With an indicator for the two top classes (1 = “Elite or Established Middle Class,” 0 otherwise), which is identical to *HighSES*, significance disappears (*χ*^2^(1) = 1.46, *p* = .227, Nagelkerke *R*^2^ = .009). Finally, only for *NAW* in Trip does a similar robustness check lead to one statistically significant result. I here defined two new dummies, one for New Affluent Workers and the next higher group (so 1 = “New Affluent Workers or Technical Middle Class,” 0 otherwise), and one for New Affluent Workers and the next lower group (1 = “New Affluent Workers or Traditional Working Class,” 0 otherwise). (This option was not available in the cases of *Elite* and *Pre*, which are limit SES classes.) For the former, the model is not significant (*χ*^2^(1) = 2.46, *p* = .117, Nagelkerke *R*^2^ = .035, 167 observations), but for the latter it is (*χ*^2^(1) = 4.85, *p* < .05, Nagelkerke *R*^2^ = .009.) All in all, however, these additional tests are hardly indicative of a more general pattern of SES influencing knowledge attribution judgments.

#### Estimating education effects

**Fig. 5:**
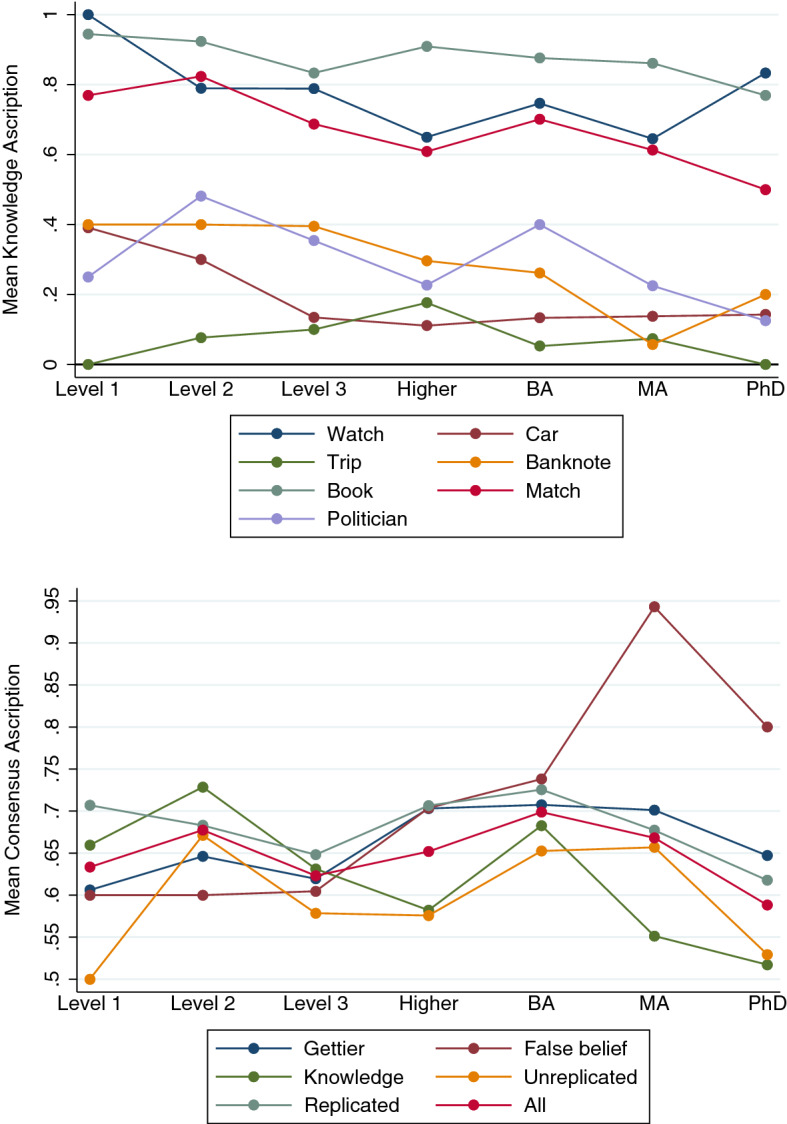
Knowledge Ascription and Education. The graphs in the upper panel plot the mean knowledge ascription for each of the seven educational categories, per vignette (*n* = 1,664). Watch, Car, and Trip are Gettier cases. Banknote is a false belief case. Book, Match, and Politician are knowledge cases. The graphs in the lower panel plot the mean consensus ascription for each of the seven educational categories, for various groups of vignettes. Consensus ascription is 1 if the knowledge/belief question was answered in line with epistemological consensus, and 0 otherwise. Scale is from .50 to .95. Gettier (*n* = 629) includes Watch, Car, and Trip. False belief (*n* = 224) includes Banknote. Knowledge (*n* = 811) includes Book, Match, and Politician. Unreplicated (*n* = 647) includes Banknote, Trip, and Politician. Replicated (*n* = 1,017) includes Watch, Book, Car, and Match. All (*n* = 1,664) includes all seven vignettes. Level 1 = “GCSE/O level below grade C, qualifications at level 1 or below,” Level 2 = “GCSE/O level grade A*–C, vocational level 2, or equivalent,” Level 3 = “A level, vocational level 3, or equivalent,” Higher = “Higher education below degree level,” BA = “Bachelor’s degree or equivalent level,” MA = “Master’s degree or equivalent level,” PhD = “Doctoral degree or equivalent level”

The measure of SES, the GBCS, is distinct from educational achievement. The results obtained by Weinberg et al. (2001) and Seyedsayamdost (2014b), by contrast, are based on measures capturing SES through a dummy for college education. Weinberg et al. (2010) explicitly acknowledge that the SES measures equate low SES respondents with those without a college degree, and they note that some results may have arisen from a lack of understanding on the part of the participants (with lower levels of education). A straightforward question is therefore whether the results reported by these scholars should rather be interpreted as educational than SES effects. Under that interpretation, Weinberg et al. (2001) should be taken to suggest that there is a correlation between education and knowledge ascription in Gettier cases, whereas Seyedsayamdost (2014b) suggests that there isn’t. We can examine this using the same two methods from above: pairwise comparisons of knowledge ascription rates and regression analysis. To anticipate, the graphs in Fig. 5 illustrate these education results (just as Fig. 3 for SES): knowledge ascription in the individual vignettes in the upper panel, and consensus ascription in grouped vignettes in the lower panel.

*Pairwise comparisons* First, we can carry out a pairwise comparison of educational classes, as I did above for SES, on the education measure introduced above. Pairwise comparison (all with Bonferroni correction) was carried out for all vignettes together, for each individual vignette, for Gettier, knowledge, replicated, and non-replicated vignettes, as above, and few statistically significant differences were found.^16^

Only a pairwise comparison between respondents with a master’s degree or equivalent level (94% of whom give the consensus answer) and respondents with an A level, vocational level 3, or equivalent (60% of whom give the consensus answer) revealed a statistically significant difference (*p* < .05), in Banknote (a false belief case).

*Regressions* One may criticize the use of pairwise comparisons on the ground that the hypothesis of interest is that higher education is associated with greater conformance with epistemological orthodoxy, and that therefore education should be thought of an ordinal measure.^17^ In fact, to estimate education effects it is standard to use an ordinal measure, or a dummy for college education.

So I performed a logistic regression with the ordinal measure of education on knowledge ascription, using the following samples: all vignettes, individual vignettes, Gettier, knowledge, replicated, and non-replicated vignettes. In two of the twelve regressions statistically significant effects show up, namely, in Banknote (a false belief case, 7 percentage points decrease in knowledge ascription per higher educational group on average), and Car (a Gettier case, 4 percentage points decrease in knowledge ascription per higher educational group on average), both *p* < .01.

To bring my approach more in line with the original studies (Seyedsayamdost, 2014b; Weinberg et al., 2001), I also considered a model with a dummy variable for college education (1 for educational achievement of bachelor’s degree or above, 0 otherwise). There are no changes vis-à-vis the earlier logistic regressions here, except that the effect in Car disappears (*χ*^2^(1) = 2.31, *p* = .131, Nagelkerke *R*^2^ = .015). So only the model with Banknote (a false belief case, *χ*^2^(1) = 7.83, *p* < .01, Nagelkerke *R*^2^ = .05, explaining 72.3% of the data, 17 percentage points decrease in knowledge ascription for respondents with college degree) is statistically significant. All in all, we do not find overwhelming evidence for the claim that education influences knowledge attribution judgments in a systematic fashion.

#### Controls

I also collected information on a range of controls that may covary with knowledge ascription: gender, age, ethnicity, and religion. I conducted regressions with these variables plus SES and education. Adding these controls to the regressions allows me to examine the robustness of the findings on SES and education, and in particular to see if the effects of *Elite* in Book, *NAW* in Trip, *Pre* in Car, and education in Banknote and Car remain. With more precision, I estimated models with each of the measures of SES introduced above (that is, dummies for each of the seven classes (*Elite*, etc.), the dichotomous variables *HighSES* and *LowSES*, and the ordinal variable *SES*), plus education (ordinal and college dummy), added to the controls, so that each estimated model had precisely one SES measure, precisely one education measure, and all four controls. And this procedure was iterated for each vignette individually, and for all grouped vignettes (all vignettes, Gettier vignettes, etc.). Dummies were used for ethnicity (1 for white ethnicity, 0 otherwise), and religion (1 for no religion, 0 otherwise), next to the usual demographics of age and gender.

Findings were the following. *Elite* is no longer significant in Book (a knowledge case) with these further controls (in the model in which the ordinal education measure is used) (*p* = .05), but is significant when the college education dummy is used (*p* < .05, with membership of the Elite decreasing the probability of ascribing knowledge by 14 percentage points). *NAW* in Trip remains significant (a Gettier case, *p* < .05, with both measures of education, with membership of the New Affluent Workers increasing knowledge ascription by 12 and 13 percentage points, respectively). *Pre* remains significant in Car (a Gettier case, *p* < .05, with a 16 percentage points increase in knowledge ascription, and *p* < .01 for the model with the college dummy, with a 19 percentage points increase).

The ordinal measure of education remains significant at the 1% level in Banknote (decreasing knowledge ascription by 7 percentage points, per educational level). Education remains significant in Car (at the 5% or 1% level, depending on the specific measure of SES, with a decrease of about 4 percentage points in knowledge ascription), except when SES is captured by the *Pre* dummy (then *p* = .09). The college dummy in Banknote remains significant (at the 5% or 1% level, depending on the specific measure of SES, with the possession of a college degree decreasing knowledge attribution by about 16 percentage points). It loses significance in Car (with *p* values not even close to the 5% threshold).

All in all, including controls in the models strengthens the findings: we find no evidence to the effect that SES is associated with knowledge attribution judgments, and only mixed and tentative evidence of education effects.

#### Exclusion criteria

Simmons and co-authors (2011) provide a set of recommendations to authors that are meant to decrease the rate of reported false positives.^18^ In this and the following study, I follow all of their recommendations. Two of them are relevant to spell out in the context of estimating the models: (i) for any control variable used in the study also to report the results without the control variable (I did this, as I estimated models with SES and education as the only independent variable); and (ii) if any observations are left out, to report all statistical results for the entire sample. As so far I have reported results based on the sample we get by using Starmans and Friedman’s (2012) exclusion criteria, I will now report results for the entire sample (2,824 observations). The Supplementary Materials provide further information.

I conducted pairwise comparisons of knowledge ascription across SES groups in this large sample. In Book (a knowledge case), Elite (92% knowledge ascription) is different from Established Middle Class (88%) and Traditional Working Class (88%) (both *p* < .05, with Bonferroni correction as explained above). Without Bonferroni correction, I find statistically significant differences in a few additional cases, such as in Banknote (a false belief case), where Established Middle Class (35% knowledge ascription) is different from Technical Middle Class (15%) (*p* < .05), and in Car (a Gettier case), where Precariat (23% knowledge ascription) and Traditional Working Class (15%) are different (*p* < .05).

In the logistic models, *Elite* in Book remains significant (*p* < .01, no controls throughout, 17 percentage points decrease in knowledge ascription). *TMC* becomes significant in Banknote (a false belief case, *p* < .05, 17 percentage points decrease). *NAW* in Trip (*p* = .67) and *Pre* in Car (*p* = .077) are no longer significant.

Turning to education, pairwise comparison of knowledge ascription across educational levels becomes a bit more outspoken than in the original sample, in such vignettes as Banknote (a false belief case) with 48% of respondents with Level 2 (GCSE/O level grade A*–C, vocational level 2, or equivalent) attributing knowledge as opposed to 13% of respondents with a master’s degree (*p* < .01, with Bonferroni, as explained above).

Perhaps a more substantial change can be seen, however, when we consider the logistic models with the ordinal measure of education; they are significant in Watch (Gettier, *χ*^2^(1) = 7.29, *p* < .05, 3 percentage points decrease of knowledge ascription per educational level), Banknote (false belief, *χ*^2^(1) = 17.81, *p* < .001, 7 percentage points decrease), Book (knowledge, *χ*^2^(1) = 4.89, *p* < .05, 2 percentage points decrease), Car (Gettier, *χ*^2^(1) = 3.96, *p* < .05, 2 percentage points decrease), Match (knowledge, *χ*^2^(1) = 6.72, *p* < .05, 4 percentage points decrease), and in the collection of knowledge vignettes (*χ*^2^(1) = 9.16, *p* < .01, 3 percentage points decrease in knowledge ascription). Using the college education dummy, the only educational effect that remains is in Banknote (*χ*^2^(1) = 13.01, *p* < .001, with respondents with a college degree being 17 percentage points less likely to attribute knowledge).

Adding controls only makes a small difference in scattered cases. The model with the dummy *EMC* (*p* < .05) and the model with the *HighSES* dummy (*p* < .05, both for the sample containing all vignettes) look a bit different from what we get if we consider individual vignettes. For then statistically significant predictors appear at 5% or 1% levels for education in Watch (with any measure of SES), Book (except *Elite*, *LowSES*, *SES*), Banknote (all groups, and all with *p* < .001), Car (except *Elite*, *TMC*, *ESW*, *Pre*, *SES*), Match (all), and Politician (except *EMC*, *NAW*, *TWC*, *ESW*, *HighSES*). College education is only significant in the model with *HighSES* (*p* < .05), all vignettes. With controls added, the college education dummy is significant at the 5% or 1% level in Banknote (any measure of SES) and Watch (with *EMC* and *NAW*).

These robustness checks cast no doubt on my claims about SES. They may, however, be taken to suggest that my findings about education should be interpreted with some care: perhaps there is a smallish education effect after all. This impression is reinforced by the fact that the pilot study, which I report later in the paper (Study 3), provides some evidence for education effects. As I did not use comprehension questions in the pilot, one possibility is that the comprehension checks have disproportionately affected particular educational categories/groups in the current study (Study 1). To examine this potential effect, I first compared observations that satisfied all criteria for inclusion as per Starmans and Friedman (2012) (1,664 observations) with those observations that were removed *solely* because of failed comprehension tests (381) (that is, had they passed the comprehension check they would have been included). The excluded subjects were not significantly different in age (*p* = .083), gender (*p* = .709), and SES (*p* = .239), but slightly more religious (46% religious vs. 40% religious, *p* < .05), more frequently not of white ethnicity (86% white vs. 91% white, *p* < .01), and less highly educated (mean educational achievement level 4.02 vs. 4.20, *p* < .05). Some selection bias in the final sample cannot therefore be excluded. Consensus knowledge ascription (55% vs. 67%, *p* < .001) was indeed significantly different between the two groups.

As a further robustness check, I estimated a model in which I added the number of comprehension check questions that are correctly answered to the analysis as a control variable, next to all other controls.

When we consider all vignettes together, the number of correctly answered comprehension questions is the only statistically significant predictor of consensus knowledge ascription (*p* < .001, except when the model has *EMC* or *HighSES* as SES measures, where *p* < .05). Moving on to consider vignettes individually, comprehension remains significant in hardly any case, and education becomes a significant predictor in some cases, albeit somewhat dependent on the exact SES variable used. With *SES* as the SES variable (so next to all other controls), education is statistically significant in Watch (Gettier, *p* < .05, 4 percentage points decrease of knowledge ascription), Banknote (false belief, *p* < .001, 6 percentage points decrease), Match (knowledge, *p* < .05, 4 percentage points decrease), and Politician (knowledge, *p* < .05, 4 percentage points decrease). These effects disappear, however, when instead of the fine-grained ordinal education measure a dummy variable for college education is used.

One possibility is that education does not so much determine the specific answer that a subject gives to a knowledge attribution question per se, but rather that subjects with higher levels of education are on average more likely to have an adequate understanding of the relevant details of the case, as specified in the vignette. But if that is true, it is difficult to explain why we find that some of the models are in the wrong direction, that is: sometimes respondents with higher levels of education tend to answer against epistemological consensus. On the other hand, we do see some effect by regressing education on the performance in the comprehension checks. I used a strong performance measure (all comprehension questions correct) and a weaker measure in which a potentially contested comprehension question was omitted (this is discussed in the Supplementary Materials). With both measures, there is a significant effect of education on comprehension with the fine-grained education measure (*p* < .01), but not if we use the college education dummy.

I think all these robustness checks have to interpreted conservatively, and should be taken as underlining the conclusion that the data do not allow us to claim that education as such influences knowledge attribution judgments. But I do believe they indicate that education requires future research attention.

This is underscored by the findings of the next studies.

## Study 2

While the Great British Class Survey was initially developed as an instrument to measure SES in the United Kingdom, its methodology is general enough to be useful to study SES in different countries. Two studies have successfully done this, for Hungary (Albert et al., 2018) and Australia (Sheppard & Biddle, 2017), but unfortunately, at the time of writing this paper, not for the United States. I therefore applied the strategies of these two studies, and developed an SES scale for the US.

To be sure, my approach has its limits. I am confident to have gathered sufficient information (through the questionnaire) to estimate reliable posterior probabilities of class membership for respondents (for here I followed the Hungary and Australia studies, and the original UK study). But the questionnaire did not contain the (large number of) items that would have been necessary if it had been my aim to come up with a sociologically rich enough description of the various classes. Adding these items (about topics such as place of birth, college attended, professional status, work environment, family composition, newspaper subscriptions, and much more) would have made the questionnaire much too long for the average MTurker. For current purposes, that wasn’t necessary, however, as I am not interested in the specific characteristics of each individual SES group, but rather in potential patterns across all SES groups with respect to knowledge attribution judgments.

To recall, SES is measured by means of six variables: income and assets, to capture economic capital; the number and mean status of one’s acquaintances, for social capital; and the number of high and emerging culture activities one engages in, for cultural capital. In their studies of Hungary and Australia, respectively, Albert et al. (2018) and Sheppard and Biddle (2017) use the same variables as in the GBCS for income and assets, which means that they split assets in two categories: the value of one’s house, and the value of one’s savings. I follow this, with the note that I change the brackets/income categories and terminology to make the questionnaire consistent with US statistical conventions.

To obtain the two variables for social capital, the researcher has to provide respondents with a list of occupations, with the instruction that they have to select an occupation if they know someone socially who has that occupation. As mentioned earlier, the list of occupations is determined on the basis of a procedure informed by the position generator approach, due to Lin (2001), which can be seen as a measure of the social status of particular occupations. Since the social status attributed to an occupation may differ across countries, using the GBCS list of occupations may not be justified outside the UK, and using the weights assigned to the positions is even less justifiable (the GCBS uses the Cambridge Social Interaction and Stratification Scale). Nevertheless, the Australia study sticks to a subset of the GBCS list. The Hungary study, by contrast, uses a different list of occupations, based on earlier Hungarian position generator research.

We are in the fortunate position that the researcher behind the position generator approach has developed a list of occupations specifically for the US (Lin, 2001). It includes precisely the following occupations: elementary school teacher, lawyer, salesperson, waiter/waitress or bartender, engineer, secretary, manager, small business owner, insurance agent, janitor, mechanic or repairman, laborer, foreman, and skilled worker. Lin also provides us with the weights (social status) that we should attach to these occupations. I include all these occupations in the questionnaire, and use Lin’s weight assignment.

Cultural capital is measured by asking respondents to what extent they engage in particular activities, or have particular music or food preferences. Partly informed by prior research, Savage and his colleagues (2013) developed an initial list of typically highbrow and emerging cultural activities. As we saw, they use multiple correspondence analysis to derive two subsets of this list, one representing highbrow cultural, and the other emerging cultural activities and music or food preferences.

Both the Australia and the Hungary study replicate this procedure somewhat, even though the basic list of activities and preferences is smaller than the original GBCS, and next to multiple correspondence analysis also principal axis factor analysis and principal component analysis are used to independently validate the scale. In the end, however, both studies arrive at highbrow and emerging cultural capital variables that are almost fully identical to those used in the GBCS.

There is no established list of highbrow and emerging cultural activities and preferences for the US, to my knowledge, and that is why I follow the Australia and Hungary study, and use the following two lists. For highbrow items: reading books, going to classical music concerts, going to the opera, going to museums, visiting historic sites, going to the theater/musical, and a preference for classical music. For emerging culture: playing computer games, surfing the internet, participating in online social networks, playing sports, watching sports, entertaining guests at home, going to a sportsclub, a preference for rock/indie, and a preference for urban/hip-hop/rap. The questionnaire included a few additional items, which based on my reading of some of the literature on class in the US might be expected of relevance in the US context (such as tennis and gourmet cooking), but multiple correspondence analysis did not confirm this. The Supplementary Materials contain the full list.

To construct the SES classes, the Australia and Hungary studies conduct a Latent Class Analysis (LCA), just as in the original GBCS study. To determine the optimal structure, researchers use a combination of formal statistical criteria (such as the Akaike (AIC) or Bayesian (BIC) information criterion), and theoretical criteria (derived from sociological work on SES). In the GBCS study, a seven-class model was chosen, despite the fact that the eight-class model had a slightly lower BIC. In the Hungary study, the lowest BIC was a twelve-class model, but the classes end up being very small if BIC decreases, and the improvement in BIC gained by adding more classes was marginal. That is why Albert and colleagues (2018) chose an eight-class model. The Australia study conducted LCA with three to seven classes, and Sheppard and Biddle (2017) chose the six-class model based on the fact that is has the lowest AIC (despite the four-class model having the lowest BIC).

Class construction was determined by similar considerations. I estimated models for six up to 13 classes. AIC is lowest at the 11-class model (11,490.18), and BIC is lowest at the 12-class model (11,932.58), which is the same as in the Hungary study, but just as in that study, these models contain classes that are too small to be relevant. In the 12-class model, for instance, only one class has more than 100 members (out of a total of 974 observations). The Hungary study therefore opted for eight classes. Unlike the Hungary model, my eight-class model still contains one small class, so I decided to work with a seven-class model. This model has an AIC of 12,469.52, a BIC of 12,908.85, and a log-likelihood of 6,144.76. These analyses were carried out on the unweighted sample. I also assigned weights to the sample derived from entropy balancing techniques used to increase representativeness (Hainmueller & Xu, 2013), with moments drawn from Pew Research Center Religious Landscape Study 2019 (for religion) and the 2018 and 2019 Unites States Census Bureau data (for all other controls), but this did not lead to different insights.

### Method

Prior power calculation in the UK study was a fairly straightforward matter. I knew that the measure of SES entailed seven classes, because the GBCS has seven classes, and hence I used that number in calculations. Moreover, it was unimportant to consider the sample size needed to robustly assign posterior SES probabilities to respondents, because I could combine my UK data with the original data from the GBCS, a procedure detailed in the Supplementary Materials.

Such a line of reasoning was unavailable in the context of the US study, however, because no measure of SES in the US has been developed using the machinery of the GBCS. So I didn’t know the exact number of SES groups prior to the experiment. However, the fact that my sample size should be large enough to allow us to run LCA as per Savage et al.’s (2013) specifications, gave help here. I reasoned that the maximum number of SES groups would be nine, as the Hungary study had shown that models with more than nine classes will tend to become uninformative. As I use one vignette, this would require 253 observations, assuming *w* = .3, *α* = .05, and *β* = .95, as above. Yet to obtain SES posterior probabilities, we would be better positioned to have a sample size not smaller than the sample size Savage et al. (2013) use, that is, 1,026 observations. So I aimed for 1,026 observations. The choice of one vignette was motivated by budget concerns, as I estimated that the exclusion criteria would lead to a fairly drastic reduction of sample size. (For the SES assignment, most of the exclusion criteria would be unimportant, as they have to do with prior exposure to a similar experiment, having had some philosophy courses, and so on.)

In the remainder of this section, I will refer to Study 1 for some of the details in order to save space in my description of the experiment and analyses.

*Participants* One thousand one hundred and fifty-nine (557 female, mean age 42 years, US residents) were recruited, using Amazon Mechanical Turk.^19^ The questionnaire was distributed through Qualtrics. I removed unfinished tasks (94), duplicate IP addresses (28), observations with outlier completion times (89), and observations that failed the astrology/sports attention check, described above (24), leaving 974 observations for assigning SES, which is slightly less than aimed for.

For the final analysis about knowledge attribution judgment, further exclusion criteria were used, as per Starmans and Friedman (2012), namely: respondents who indicated that they had participated in similar experiments before (225 for total sample, a further 194 from the 974), those that failed one or more of four comprehension questions (462, or 352 of the 974), and observations with missing SES values (10), with a remaining sample size of 480 (245 female, mean age 42 years), which is more than the amply 250 of the above power calculations.

*SES* I first briefly discuss the SES measure, and refer to the Supplementary Materials for details. Following variable construction as per Savage et al. (2013) throughout, I defined, on the sample of 974 observations, the six variables that capture economic, social, and cultural capital. It is important to underscore again that based on the research for this study, it is impossible to confidently develop a sociologically accurate understanding of the various resulting classes as in the UK, Australia, and Hungary studies, as that would have required introducing an array of further items making the survey too long. Hence I give simple non-descriptive names to the seven classes: SES1, SES2, and so forth.

This does not of course change the way respondents are assigned to SES classes. So I can carry out most of the analyses. But it makes it a bit difficult to develop an ordinal measure of SES. The Australia and Hungary study provide an ordering of the classes simply along the lines of the dimension of economic capital only, so they do not use more information to determine the ordering than the two variables (income, assets) that capture economic capital. I could do that with the available information as well of course, but find that this goes somewhat against the sense of the GBCS, and also prefer a methodologically more conservative approach. So I restrict attention to categorical SES measures here, and run a separate regression with the two economic variables, for robustness.

*Materials and procedure* I now describe the experiment proper. The experiment involved one vignette (Watch, a Gettier case), drawn from Starmans and Friedman (2012). The question was: “Which of the following is true?” with possible answers in random order: “Peter really knows that there is a watch on the table,” and “Peter only thinks that there is a watch on the table.” Standard demographic questions were asked (age, gender, etc.).

The questionnaire was identical to the one used in the UK study, with the exception of the SES questions being adjusted to the US context (different brackets for economic variables, and different occupations, activities, and preferences, as explained above). This also applies to the education measure. Comprehension checks, questions about prior exposure to philosophy and similar experiments, the astrology/sports attention check, and three unrelated questions about the Covid-19 pandemic and political identity, for use in another study and presented at the end of the survey, were also included. No other items were used. The Supplementary Materials provide details.

### Results

#### Replication of published study

WKA was different from chance (*M* = 4.89, *SD* = .31, *t*(489) = 15.64, *p* < .001), as was the dichotomous question (binomial, *p* < .001, two-sided), with 77% of respondents attributing knowledge to the protagonist of the vignette. I replicated Starmans and Friedman’s (2012) original study (*Z* = .644, *p* = .520, two-sided difference in proportion test/z test, 72% of 47 respondents in the original study, 77% of 490 respondents here).

#### Estimations

*SES* Pairwise comparison of knowledge ascription rates across the seven SES classes did not reveal statistically significant differences between classes (Bonferroni corrected, as explained in Study 1). Only two SES classes (SES6 and SES7) turn out significantly different if we do not adjust for multiple comparisons (*p* < .05).

Logistic regressions with dummies for SES1, SES2, and so on as independent variables are not significant, with *p* values far away from the 5% threshold, except for SES6 (*χ*^2^(1) = .04, *p* = .081, Nagelkerke *R*^2^ = .010). The power of these tests was high (1.0), assuming a moderate effect size (*w* = .3) and setting *α* = .05.

**Fig. 6:**
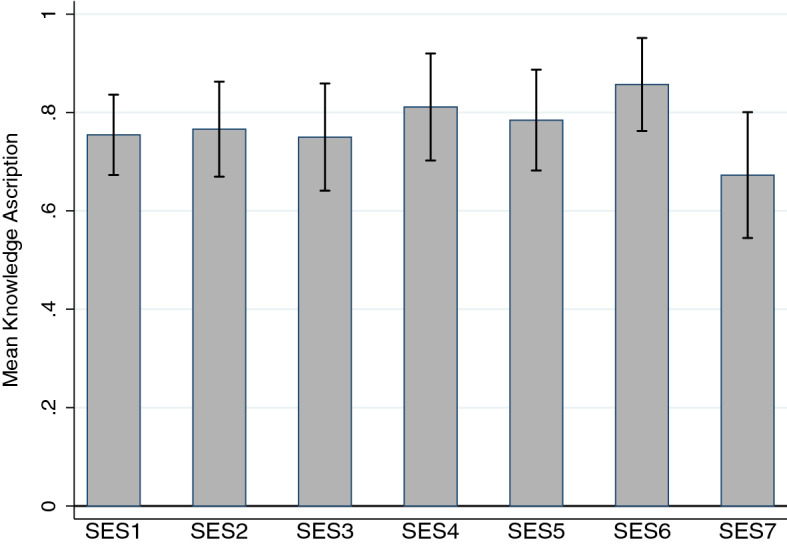
Knowledge Ascription per SES Group (Study 2) *n* = 490. The figure displays the mean knowledge ascription per SES group, of Watch (a Gettier case), which was significantly different from chance everywhere (see main text). Error bars indicate 95% confidence intervals. The order of the SES groups is arbitrary (see main text)

Using a categorical variable for SES (so 1 = “SES1,” 2 = “SES2,” etc.) did not change the findings (*χ*^2^(6) = 6.20, *p* = .401). A further analysis using an ordinal measure of SES is something that the data do not allow us to do, as motivated above. We must conclude, then, that SES and knowledge ascription in this Gettier case are not correlated. Figure 6 displays knowledge ascription for each of the seven SES groups.

**Fig. 7:**
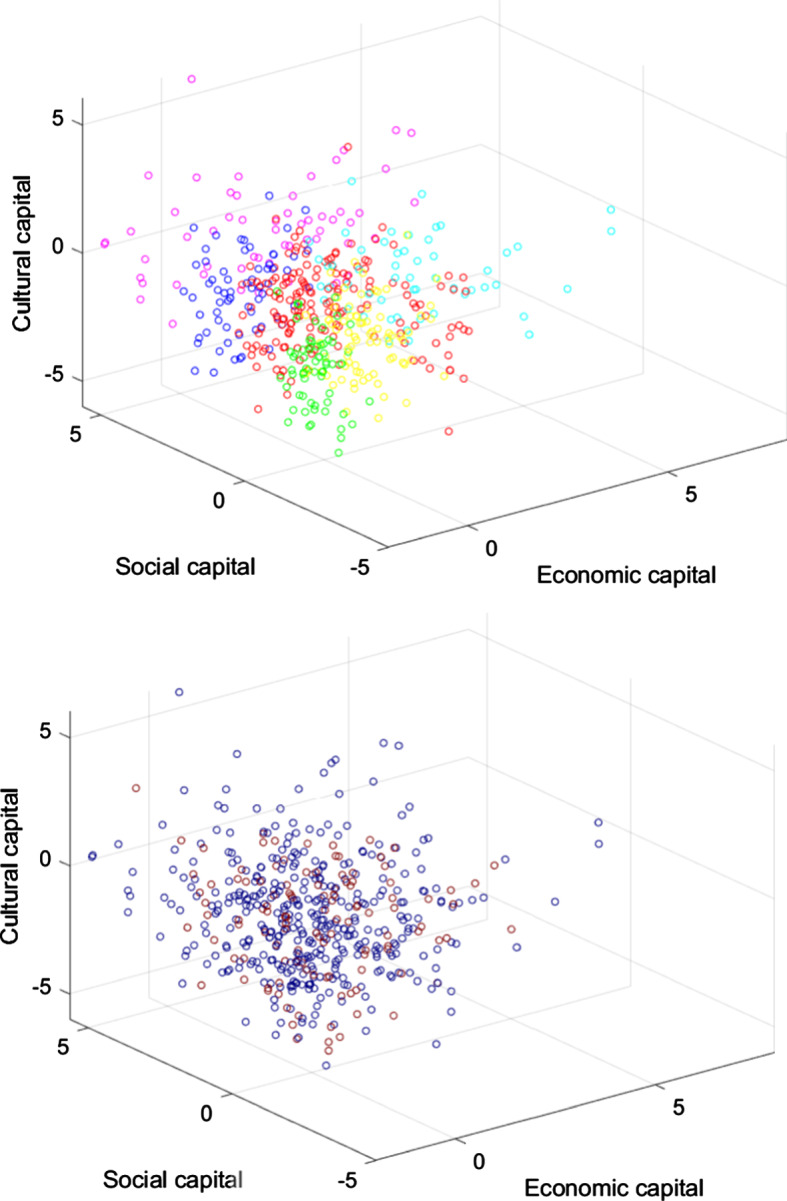
Economic, Social, and Cultural Capital, SES Group Membership, and Knowledge Ascription (Study 2) *n* = 490. The graphs plot each subject in three-dimensional space. Economic capital is measured by the sum of income and assets. Social capital is the sum of the mean status of one’s social contacts and the number of one’s social contacts. Cultural capital is the sum of the high and emerging culture scores. For the six underlying variables standardized versions are used. In the upper panel, the color of each subject represents the SES group to which they are assigned using Latent Class Analysis as per Savage et al. (2013). Orange = “Elite,” yellow = “Established Middle Class,” green = “Technical Middle Class,” blue = “New Affluent Workers,” indigo = “Traditional Working Class,” violet = “Emergent Service Workers,” and red = “Precariat.” In the lower panel, the color of each subject represents whether they attributed knowledge to the protagonist of Watch (a Gettier case) (blue) or not (red). No noise is added to observations to duplicate coinciding observations

As announced above, the Australia and Hungary studies introduce an ordinal measure of SES, on the basis of economic capital. We could do that too, as I said above, but I find it methodologically preferable to estimate a model with these two variables and/or an aggregated economic capital variable, without the social and cultural capital measures. So I also ran a logistic regression on the two economic variables (standardized versions of income and assets). But this does not radically change the views articulated above. Income is not significant, while assets barely hits significance at *p* = .049, with the interpretation that one standard deviation of assets increases knowledge attribution in Watch by 4 percentage points, and when they are combined (sum both variables) to capture economic capital as an aggregated concept, significance is lost again (*p* = .688). This means that there is not even a robust sense in which one component of SES, namely economic capital, would predict judgment in this knowledge attribution task. Figure 7 illustrates the results by contrasting the clustered SES groups, plotted in three-dimensional (economic, social, cultural capital) in the upper panel, with knowledge ascription in the lower panel, in the same way as in the previous section. Table 2 in the Appendix contains further regression details.

*Education* To estimate education effects, I began with pairwise comparisons across educational classes. With Bonferroni correction, applied as explained in Study 1, two classes are different at the 5% level, namely, respondents with some college but no degree (85% knowledge attribution), and respondents with a bachelor’s degree (70% knowledge attribution). Without Bonferroni, only one additional pair becomes significantly different (respondents with a high school degree and no college (86%), and respondents with a bachelor’s degree (70%)).

**Fig. 8:**
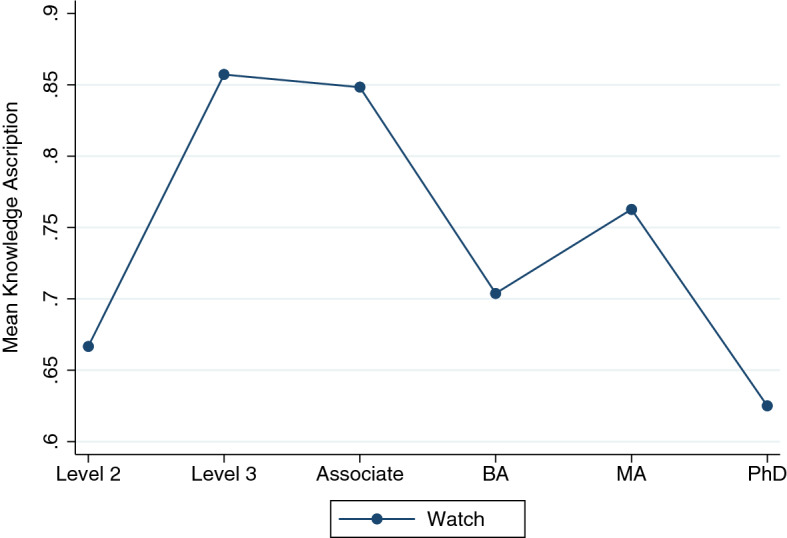
Knowledge Ascription and Education (Study 2) *n* = 490. The figure plots the mean knowledge ascription against educational achievement, of Watch (a Gettier case). Scale is from .60 to .90. Level 1 = “Less than 9th grade” (no observations, omitted), Level 2 = “9th to 12th grade, no diploma,” Level 3 = “High school graduate,” Associate = “Some college (but no degree), or associate’s degree or equivalent level,” BA = “Bachelor’s degree or equivalent level,” MA = “Master’s degree or equivalent level,” PhD = “Doctoral degree or equivalent level”

A logistic regression, exploiting the ordering of the independent variable (which in the case of education is natural), reveals a significant impact of education on knowledge ascription, at the 5% level, with each educational level on average decreasing the probability of attributing knowledge by 6 percentage points. When we ignore the ordering of the educational groups this effect remains (*χ*^2^(5) = 13.47, *p* < .05, Nagelkerke *R*^2^ = .042, explaining 76.5% of the data). Using a dummy for education raises significance to the 0.1% level, with a college graduate 13 percentage points less likely to attribute knowledge. Figure 8 plots knowledge ascription against education.

*Controls* Next I used the full array of control variables in the regression, so I conducted a regression with SES and education, plus the remaining independent variables gender, age, ethnicity, and religion. Here as in the UK study, I conducted regressions with various measures for SES and education. For SES, I introduced dummies for each of the seven SES groups. For education, I used the ordinal measure, and a dummy for college education. With all these controls in place, education is significant at the 1% level, and college at the 0.1% level, irrespective of the variable used for SES, and roughly with size as above. With the exception of SES7, no SES variable is significantly associated with knowledge ascription. We get fairly strong evidence of an effect of education on knowledge ascription here, then, but again no evidence for SES effects. As none of the SES variables used here are ordinal, I also estimated models with economic variables. Here assets turn out to be significantly associated with knowledge attribution judgments (*p* < .05, with about 3 percentage points higher likelihood of knowledge ascription per standard deviation).

*Exclusion criteria* As in the previous study, I followed Simmons et al. (2011), and replicated all results with the full sample. Some methodological issues turn up here. The full sample contains observations with missing items, which makes SES assignment impossible. I therefore use the largest sample that includes only observations with SES assignments. This sample has size 974.

WKA is different from chance (*M* = 4.85, *SD* = .23, *t*(973) = 21.33, *p* < .001), and the dichotomous question is also different from chance (binomial, *p* < .001, two-sided), with 76% of respondents assigning knowledge. Starmans and Friedman’s (2012) experiment is replicated (*Z* = 0.65, *p* = .514, two-sided difference in proportion test/z test). Across SES groups, there are no significant pairwise differences (Bonferroni corrected, as explained in Study 1, and also unadjusted). In none of the regressions (without controls) is SES significant. Pairwise comparison (Bonferroni corrected) does not detect any significant differences between educational groups. Unadjusted for multiple comparisons, two pairs are different at the 1% level. Without controls, education is significantly associated with knowledge ascription (*χ*^2^(1) = 7.50, *p* < .01, with one level up decreasing knowledge ascription by 4 percentage points), also if the ordering of educational groups is suppressed (*χ*^2^(5) = 12.95, *p* < .05, Nagelkerke *R*^2^ = .020, explaining 76.5% of the data points). With the college education dummy significance reaches the 1% level, with a college degree decreasing knowledge ascription by 9 percentage points.

With controls added (as described above), education is significant at the 1% level irrespective of the SES variables used. This is also true if the college education dummy is used. I note for completeness that age and gender turn out significant at the 1% level in most of these regressions as well, but with much smaller odds ratios than education.

In Study 1, I also considered whether some education effects are driven by the fact that subjects with higher levels of education on average tend to understand the vignettes better. No such effects are found here, as education and passing the comprehension checks are not significantly correlated, both for the fine-grained and the college dummy variable.

## Study 3

I briefly describe the pilot experiment that started the investigations. This study was carried out as part of a larger study on the effects on knowledge attribution judgments of learning and other environmental factors (de Bruin, 2021). I used the same seven vignettes as in Study 1, and sourced a UK sample using the same online platform, Prolific. I had to restrict the number of GBCS items in order to keep the length/duration of the experiment within acceptable limits. Unlike in Study 1 and 2, I did not ask comprehension questions, because this would have potentially conflated the results on learning and other environmental factors in the larger experiment. The sample size of the experiment was not determined by prior power considerations targeted to the pilot reported here. The Supplementary Materials contain details about the sample (such as exclusion criteria) and variable construction as well as figures.

### Method

*Participants* Eight hundred and twenty participants (385 female, mean age 38 years, UK residents) were recruited using Prolific. The questionnaire was distributed through Qualtrics. I removed unfinished tasks (43), duplicate IP addresses (79), and observations with completion times less than 60 or more than 1,000 seconds (43), and obtained a sample of 709 participants (361 female, mean age 38 years). I paid participants the Prolific rate of £0.50 ($0.65) per 5 min of their time.

*Materials and procedure* The experiment involved the same seven vignettes used in Study 1. Questions from the GBCS were included. I did not include the full range of questions, but restricted myself to the set of items that can be found on the BBC website, as the GBCS is a collaboration between Savage and colleagues and the BBC.^20^ The income, house value, and remaining assets brackets are slightly less fine-grained than in Study 1. The variables for social capital included precisely the following occupations: secretary, nurse, teacher, cleaner, university lecturer, artist, electrician, office manager, solicitor, farm worker, chief executive, software designer, call center worker, postal worker, scientist, lorry driver, accountant, shop assistant. The cultural capital items were: go to stately homes, go to the opera, listen to jazz, listen to rock/indie, go to gigs, play video games, watch sports, go to the theater, exercise/go to gym, use Facebook/Twitter, socialize at home, go to museums/galleries, listen to classical music, do arts and crafts, watch dance or ballet, listen to hip-hop/rap.

Education was captured using the measure from Study 1, and the same demographic variables were included here.

### Results

#### Replication of published studies

For three cases (Watch, Banknote, Book) I calculated WKA, which was significantly different from chance in Watch (Gettier, *M* = 3.47, *SD* = 7.29, *t*(131) = 5.48, *p* < .001, all two-sided) and Book (knowledge, *M* = 4.23, *SD* = 6.97, *t*(127) = 6.87, *p* < .001), but not in Banknote (false belief, *M* = .70, *SD* = 7.93, *t*(126) = 1.00, *p* = .321). Confidence ratings were not available for the other vignettes, as no items were included in the survey, so no WKA measure could be developed for them.

Considering dichotomous knowledge ascription, rates are significantly different from chance (binomial, *p* < .001, all two-sided) in Watch (Gettier, 72%), Book (knowledge, 74%), Car (Gettier, 25%), Trip (Gettier, 5%), and Politician (knowledge, 30%), but not in Banknote (false belief, 43%, *p* = .155) and Match (63%, *p* = .015).

I replicated Watch (*Z* = .05, *p* = .961, two-sided difference in proportion test/z test, 72% of 47 respondents in the original study ascribing knowledge, 72% of 132 respondents here), Trip (*Z* = 1.89, *p* = .059, 39% of 64 respondents in the original study, 26% of 128 respondents here), and Politician (*Z* = 1.22, *p* = .222, 65% of 60 respondents, 75% of 75 respondents here), but obtained different results in Banknote (*Z* = 3.96, *p* < .001, 11% of 46 respondents, 43% of 127 respondents here), Book (*Z* = 2.05, *p* < .05, 88% of 51 respondents, 74% of 128 respondents here), Car (*Z* = 7.41, *p* < .001, 14% of 58 respondents, 72% of 132 respondents here), and Match (*Z* = 2.45, *p* < .05, 78% of 41 respondents, 57% of 127 respondents here), relative to earlier studies referenced in the discussion of Study 1.

#### Estimations

*SES* Pairwise comparison of consensus ascription rates across the seven SES classes did not reveal statistically significant differences between classes (Bonferroni corrected, as explained in Study 1). Without adjustment, Technical Middle Class (44%) is significantly different from three of the other six classes, namely, Established Middle Class (60%), Emergent Service Workers (59%), and Precariat (62%), all *p* < .05. Considering each vignette individually, only one pair in Match is different (Emergent Service Workers (78%) vs. New Affluent Workers (20%), *p* < .05, Bonferroni adjusted). Without correcting for multiple comparisons, the significance increases naturally (*p* < .01), and two extra pairs reach significance.^21^ Restricting ourselves to Gettier vignettes, or to the knowledge vignettes, none of the pairwise differences is significant with Bonferroni. Without Bonferroni adjustment, this goes through for Gettier, but not for knowledge, where two pairs are different.^22^

With the exception of the model with the dummy *TMC*, no logistic regression (without controls) surpasses the 5% significance level (*χ*^2^(1) = 6.72, *p* < .01, Nagelkerke *R*^2^ = .013, explaining 59.2% of data). A chi-square test with *SES* as a categorical variable yields *χ*^2^(6) = 7.30, *p* = .294, with power 1.0, assuming *w* = .3 and *α* = .95, all on all vignettes together.

Restricting the analysis to individual vignettes yields significant results only with the *HighSES* dummy in Banknote (*χ*^2^(1) = 5.33, *p* < .0), with power of 92% (127 observations, assumptions as per above), with *TMC* in Book (*χ*^2^(1) = 5.95, *p* < .05), power 92% (128 observations), and with *NAW* (*χ*^2^(1) = 5.12, *p* < .05) and *LowSES* (*χ*^2^(1) = 8.91, *p* < .01) in Match, power 81% (90 observations). Table 3 in the Appendix contains further information.

*Education* Pairwise comparison of educational levels (Bonferroni adjusted, as explained in Study 1) detects a difference between respondents with master’s degrees (70% consensus ascription) and respondents with the lowest educational attainment (45% consensus ascription), *p* < .05, all vignettes. These two groups also differ significantly (with Bonferroni) in the Gettier subsample (*p* < .05), with knowledge (not: consensus) ascription rates of 63% (lowest educational bracket) and 25% (master’s degree).

A logistic regression taking into account the obvious ordering of educational achievement categories suggests a connection with consensus knowledge ascription on all vignettes together (*χ*^2^(1) = 7.57, *p* < .01). These effects are, however, unevenly distributed across individual vignettes, as only Watch (Gettier, *p* < .01) and Banknote (false belief, *p* < .05) show significance here if considered individually. If we take all observations of all Gettier vignettes together, the model is significant at the 5% level.

*Controls* What happens to SES and to education if we add controls? With gender, age, ethnicity, religion, education, and the ten different variables for SES (each model with exactly one SES variable), education is significant in the expected direction in all models at the 5% and sometimes at the 1% level, on the entire sample of all vignettes. SES, by contrast, is significant for none of the measures, except for the *TMC* dummy (*p* < .01).

Looking at individual vignettes (running again ten regressions for each of the SES variables), only *TMC* is significant in Book (knowledge, *p* < .05), *NAW* in Watch (Gettier, *p* < .01), and *LowSES* in Match (knowledge, *p* < .05). Education reaches the 5% level in some of the models (Watch, with all ten SES variables), Banknote (false belief, with *TMC*), Politician (Knoweldge, with *LowSES* and *SES*).

Considering types of vignettes as in Study 1 (Gettier, false belief, etc.), only one model is significant for SES, namely, in knowledge vignettes using the *TMC* dummy, at the 5% level. Education is significant at the 5% or 1% level for Gettier vignettes with all models, except the one with *Elite* as SES measure.

## Conclusion

A pioneering paper in the field of experimental philosophy defended two empirical claims: that people of different cultures, and that people with different socioeconomic status (SES) respond differently to questions about Gettier vignettes (Weinberg et al., 2001). The claim about culture has attracted quite a bit of attention, with several above referenced studies giving evidence that the Gettier intuition may actually be shared across cultures to the same degree. The claim about SES has received far less attention. One reason may have to do with experimental design. It is fairly easy to gather cross-cultural data by fielding your questionnaires in different countries, or in multicultural societies such as the US, but it is far less straightforward to gather data across different socioeconomic classes because, up to very recently, there was no unambiguous measure for SES. That is why empirical researchers in the social sciences used income, wealth, or education as a proxy for SES; with clear drawbacks, for no longer is it then possible to distinguish education and economic effects from class effects.

In this paper, I used a novel instrument to measure SES: the Great British Class Survey, developed by Mike Savage and his colleagues (2013). This measure is independent of education. And while it contains income and wealth to measure respondents’ economic capital, it also contains four other variables measuring respondents’ social and cultural capital.

Using this measure, I found no evidence for class effects on Gettier judgments, nor for effects on knowledge attribution tasks more generally (tasks involving false beliefs, and tasks involving vignettes in which there is knowledge on an orthodox understanding).

I believe that these results are sufficiently stable. To begin with, I investigated knowledge attribution judgments in a society (the United Kingdom, using Prolific) in which class arguably plays a more distinct role than in many other Western countries, and I used a measurement instrument specifically developed and validated in the UK. I in fact ran two studies, one using comprehension questions (Study 1), and the other not (Study 3, the pilot), and they deliver the same results. I also conducted a separate experiment in the United States, on a different online portal (Amazon Mechanical Turk), for which I developed an SES measure following the methodology of the GBCS (the first application of the GBCS methodology to the US, to my knowledge). Secondly, I tested for robustness by including various alternative measures of SES. The GBCS does not entail an ordering of the seven SES groups, at least not without further assumptions. So when I carried out a pairwise comparison of knowledge ascription rates across classes, I treated them effectively as nominal data. Weinberg et al. (2001), however, considered a dichotomous variable, distinguishing between high and low SES (which, since they use education as a proxy for SES, amounted to: college education, yes or no?). I replicated this with various measures of SES (but of course independent of educational achievement), which all delivered the same results. I therefore consider the results sufficiently stable.

Thirdly, I included a host of control variables in the regression, which reaffirmed the conclusion that SES is not associated with knowledge ascription judgments.

Mixed evidence for education effects was found. Studies 2 and 3 provide evidence that might suggest that higher levels of educational achievement are associated with increased conformance with epistemological consensus. Study 1 provides less support for an association of between education and consensus ascription. I report my findings therefore without making any definite claims, and suggest this question for further future research.

### Supplementary Information

Below is the link to the electronic supplementary material.

Supplementary file1 (PDF 490 kb)

## Appendices

### Appendix A: Vignettes

#### Watch

Peter is in his locked apartment reading, and is about to have a shower. He puts his book down on the coffee table, and takes off his black plastic watch and leaves it on the coffee table. Then he goes into the bathroom. As Peter’s shower begins, a burglar silently breaks into the apartment. The burglar takes Peter’s black plastic watch, replaces it with an identical black plastic watch, and then leaves. Peter is still in the shower, and did not hear anything.

Which of the following is true?

- Peter really knows that there is a watch on the table.
- Peter only believes that there is a watch on the table.

#### Banknote

Peter is in his locked apartment reading, and is about to have a shower. He puts his book down on the coffee table, and takes off his black plastic watch and leaves it on the coffee table. Then he goes into the bathroom. As Peter’s shower begins, a burglar silently breaks into the apartment. The burglar takes Peter’s black plastic watch, replaces it with a banknote, and then leaves. Peter is still in the shower, and did not hear anything.

Which of the following is true?

- Peter really knows that there is a watch on the table.
- Peter only believes that there is a watch on the table.

#### Book

Peter is in his locked apartment reading, and is about to have a shower. He puts his book down on the coffee table, and takes off his black plastic watch and leaves it on the coffee table. Then he goes into the bathroom. As Peter’s shower begins, a burglar silently breaks into the apartment. The burglar takes Peter’s black plastic watch, replaces it with an identical black plastic watch, and then leaves. Peter is still in the shower, and did not hear anything.

Which of the following is true?

- Peter really knows that there is a book on the table.
- Peter only believes that there is a book on the table.

#### Car

Bob has a friend, Jill, who has driven a Buick for many years. Bob therefore thinks that Jill drives an American car. He is not aware, however, that her Buick has recently been stolen, and he is also not aware that Jill has replaced it with a Pontiac, which is a different kind of American car.

Which of the following is true?

- Bob really knows that Jill drives an American car.
- Bob only believes that Jill drives an American car.

#### Match

A pyromaniac has just purchased a box of Sure-Fire Matches. He has done so many times before and has noted that they have always lit when struck unless they were wet. Furthermore, he knows that oxygen must be present for things to burn and that the observed regularity between the matches’ being struck and their lighting is not a mere coincidence. After perceiving that the matches are dry and that there is plenty of oxygen present, he proceeds to strike one of the matches, confident that it will light. It does.

Which of the following is true?

- The pyromaniac really knew that the match would light.
- The pyromaniac only believed that the match would light.

#### Trip

Luke works in an office with two other people, Victor and Monica. All winter Victor has been describing his plans to go to Las Vegas on his vacation, even showing Luke the website of the hotel where he has reservations. When Victor is away on vacation, Luke sees Victor’s Facebook photos of himself with Vegas landmarks in the background, together with status updates about how much he is enjoying his trip. When he gets back to work, Victor talks a lot to Luke about how much fun he had vacationing in Las Vegas. However, Victor didn’t really go on the trip; he has just been pretending. His tickets and reservations were cancelled because his credit card was maxed out, and he secretly stayed home in Markham, very skillfully faking the Facebook pictures using Photoshop. Meanwhile, Monica just spent a weekend vacationing in Las Vegas, but kept this a secret from all her co-workers.

Which of the following is true?

- Luke really knows that one of his co-workers recently vacationed in Las Vegas.
- Luke only believes that one of his co-workers recently vacationed in Las Vegas.

#### Politician

A political leader is assassinated. A reporter on the scene sends news of the assassination to her news agency so that the story can be included in the day’s final edition of the paper. Jill buys a copy of that paper and reads the story of the assassination that was dictated by the reporter who witnessed the event.

Which of the following is true?

- Jill really knows that the political leader has been assassinated.
- Jill only believes that the political leader has been assassinated.

### Appendix B: Regressions

**Table 1:** Regressions Study 1

	OR	SE	*p* value	95% CI
*All vignettes*				
SES	1.008	0.027	0.778	(0.956, 1.062)
Constant	1.932	0.235	0.000	(1.521, 2.453)
*χ*^2^ (1) = 0.08, Nagelkerke *R*^2^ = 0.000, *n* = 1,664				
Education	1.037	0.036	0.298	(0.969, 1.110)
Constant	1.713	0.264	0.000	(1.267, 2.316)
*χ*^2^ (1) = 1.08, Nagelkerke *R*^2^ = 0.001, *n* = 1,664				
Gender	1.226	0.129	0.052	(0.998, 1.506)
Age	1.147	0.079	0.046	(1.002, 1.312)
Ethnicity	1.133	0.218	0.518	(0.777, 1.651)
Religion	0.834	0.093	0.103	(0.670, 1.037)
Education	1.053	0.038	0.157	(0.980, 1.131)
SES	1.031	0.029	0.292	(0.974, 1.090)
Constant	0.759	0.279	0.453	(0.370, 1.559)
*χ*^2^ (6) = 14.86, Nagelkerke *R*^2^ = 0.012, *n* = 1,664				
*Gettier vignettes*				
SES	0.981	0.044	0.673	(0.898, 1.072)
Constant	2.239	0.464	0.000	(1.492, 3.361)
*χ*^2^ (1) = 0.18, Nagelkerke *R*^2^ = 0.000, *n* = 629				
Education	1.097	0.062	0.104	(0.981, 1.225)
Constant	1.416	0.349	0.157	(0.874, 2.294)
*χ*^2^ (1) = 2.64, Nagelkerke *R*^2^ = 0.006, *n* = 629				
Gender	1.449	0.250	0.031	(1.034, 2.031)
Age	1.117	0.123	0.317	(0.900, 1.386)
Ethnicity	0.990	0.337	0.977	(0.508, 1.931)
Religion	1.122	0.201	0.519	(0.790, 1.594)
Education	1.095	0.064	0.124	(0.976, 1.229)
SES	0.999	0.048	0.981	(0.910, 1.097)
Constant	0.593	0.358	0.387	(0.182, 1.934)
*χ*^2^ (6) = 8.75, Nagelkerke *R*^2^ = 0.019, *n* = 629				

The dependent variable is *Corr*, and captures whether the case questions were answered according to consensus. The first column reports the odds ratios (OR), the second the standard errors (SE), the third the *p* value, and the fourth the 95%-confidence interval (CI). Gender (1 = “Male,” 0 = “Female”), Ethnicity (1 = “White ethnicity,” 0 otherwise), and Religion (1 = “No religion,” 0 otherwise) are dummies. Education and SES are the ordinal measures described in the text. The first three regressions take all vignettes together. The last three concern Gettier vignettes only. The first two of each of these triples display results without controls. The third contains all controls

**Table 2:** Regressions Study 2

	OR	SE	*p* value	95% CI
*Watch vignette (Gettier)*				
Income	1.170	0.122	0.133	(0.953, 1.435)
Assets	0.786	0.096	0.049	(0.618, 0.999)
Constant	0.302	0.033	0.000	(0.244, 0.373)
*χ*^2^ (2) = 5.91, Nagelkerke *R*^2^ = 0.018, *n* = 490				
Education	1.380	0.173	0.010	(1.079, 1.764)
Constant	0.067	0.041	0.000	(0.020, 0.222)
*χ*^2^ (1) = 6.77, Nagelkerke *R*^2^ = 0.021, *n* = 490				
Gender	0.557	0.124	0.009	(0.360, 0.862)
Age	0.832	0.142	0.282	(0.596, 1.163)
Ethnicity	1.281	0.360	0.378	(0.739, 2.222)
Religion	0.874	0.199	0.553	(0.560, 1.364)
Education	1.489	0.194	0.002	(1.152, 1.923)
Income	1.180	0.128	0.127	(0.954, 1.460)
Assets	0.732	0.098	0.020	(0.563, 0.952)
Constant	0.145	0.119	0.019	(0.029, 0.723)
*χ*^2^ (7) = 23.67, Nagelkerke *R*^2^ = 0.071, *n* = 490				

The dependent variable is *Corr*, and captures whether the case question was answered according to consensus. The first column reports the odds ratios (OR), the second the standard errors (SE), the third the *p* value, and the fourth the 95%-confidence interval (CI). Gender (1 = “Male,” 0 = “Female”), Ethnicity (1 = “White ethnicity,” 0 otherwise), and Religion (1 = “No religion,” 0 otherwise) are dummies. Education is the ordinal measures described in the text. There are no ordinal SES measures, as explained in the main text, and so only results with the economic variables are reported. Income and Assets are standardized. The first two regressions display results without controls. The third contains all controls

**Table 3:** Regressions Study 3

	OR	SE	*p* value	95% CI
*All vignettes*				
SES	1.022	0.040	0.565	(0.948, 1.103)
Constant	1.258	0.223	0.195	(0.889, 1.779)
*χ*^2^ (1) = 0.33, Nagelkerke *R*^2^ = 0.001, *n* = 709				
Education	1.137	0.053	0.006	(1.037, 1.247)
Constant	0.838	0.164	0.367	(0.570, 1.231)
*χ*^2^ (1) = 7.57, Nagelkerke *R*^2^ = 0.014, *n* = 709				
Gender	0.945	0.147	0.714	(0.697, 1.281)
Age	1.039	0.126	0.750	(0.820, 1.317)
Ethnicity	0.504	0.194	0.075	(0.237, 1.072)
Religion	1.283	0.208	0.125	(0.934, 1.763)
Education	1.149	0.058	0.006	(1.041, 1.269)
SES	1.054	0.045	0.211	(0.970, 1.145)
Constant	1.066	0.659	0.918	(0.317, 3.580)
*χ*^2^ (6) = 15.21, Nagelkerke *R*^2^ = 0.029, *n* = 709				
*Gettier vignettes*				
SES	0.968	0.061	0.603	(0.855, 1.095)
Constant	1.577	0.461	0.120	(0.889, 2.797)
*χ*^2^ (1) = 0.27, Nagelkerke *R*^2^ = 0.001, *n* = 280				
Education	1.283	0.098	0.001	(1.105, 1.491)
Constant	0.521	0.166	0.041	(0.279, 0.974)
*χ*^2^ (1) = 11.00, Nagelkerke *R*^2^ = 0.052, *n* = 280				
Gender	0.934	0.235	0.787	(0.571, 1.529)
Age	1.049	0.206	0.806	(0.715, 1.541)
Ethnicity	0.359	0.239	0.124	(0.097, 1.326)
Religion	1.106	0.289	0.699	(0.663, 1.846)
Education	1.272	0.104	0.003	(1.085, 1.493)
SES	1.025	0.070	0.723	(0.896, 1.171)
Constant	1.214	1.225	0.847	(0.168, 8.765)
*χ*^2^ (6) = 14.08, Nagelkerke *R*^2^ = 0.066, *n* = 280				

The dependent variable is *Corr*, and captures whether the case questions were answered according to consensus. The first column reports the odds ratios (OR), the second the standard errors (SE), the third the *p* value, and the fourth the 95%-confidence interval (CI). Gender (1 = “Male,” 0 = “Female”), Ethnicity (1 = “White ethnicity,” 0 otherwise), and Religion (1 = “No religion,” 0 otherwise) are dummies. Education and SES are the ordinal measures described in the text. The first three regressions take all vignettes together. The last three concern Gettier vignettes only. The first two of each of these triples display results without controls. The third contains all controls

## Footnotes

1 Weinberg et al. (2001) is a cornerstone of the field. See Sytsma and Livengood (2016) for an account tracing the history of experimental philosophy back to the pre-Socratics, Descartes, Locke, and others.

2 Also see Machery (2017), which is also relevant to the expertise defense, and Machery (2021).

3 Seyedsayamdost (2014a) takes as cut-off point whether the respondent has taken one or more courses at college level, so the set of high SES individuals is a bit larger than in Weinberg et al. (2001).

4 Their methodology involved sampling among individuals hypothesized to belong to a particular SES group. For instance, sampling in Philadelphia involved recruiting passersby near the University of Pennsylvania (which delivers their high SES sample) and near a McDonald’s restaurant in West Philadelphia (their low SES sample). From an economics or sociology perspective, this sampling procedure is frankly a bit unusual. Also, I do not here count on the experiment replicating.

5 The present discussion has benefited greatly from the critical comments of two anonymous reviewers for this journal on an earlier version of this paper, who made me realize that it is difficult to get sufficient mileage out of psychological and sociological research as it stands.

6 See Savage et al. (2013) for details.

7 Assume that the latent variable $x = 1, \ldots ,n$ captures $n$ (socioeconomic, etc.) classes (in the case of GBCS, $n = 7$). Then the core idea of latent class models is expressed by the following identity: $f(y_{i} ) = \sum\nolimits_{{x = 1}}^{n} {P(x)} \times \prod\nolimits_{{t = 1}}^{k} {f(y_{{it}} |x)}$, where $f$ is a probability density function, $y_{i}$ a vector of the values of the $k$ variables for individual $i$ (with $k = 6$ in the case of GBCS), and $P\left( x \right)$ is the probability of belonging to latent class $x$. Posterior probabilities are calculated using Bayes’ theorem, capturing the probability $\pi _{{ix}} = P\left( {i \in x|y_{i} } \right)$ that $i$ is a member of $x$, given *i*’s scores on the $k$ variables $y_{{it}}$. Following the *empirical Bayes modal classification rule*, respondent $i$ is assigned to the class $x$ for which $\pi _{{ix}}$ is greatest. The model is in fact estimated for $n = 1,2, \ldots$ , and model choice is informed by Akaike’s Information Criterion (AIC) and the Bayesian Information Criterion (BIC).

8 I estimate most models with SES as a categorical variable, but some analyses use an ordering of socioeconomic classes that Savage et al. (2013) introduce.

9 Hoenig and Heisey (2001) criticize using power in the context of testing null hypotheses, and advocate other approaches such as equivalence testing. I here follow Machery (2012), who defends power analysis to infer null hypotheses.

10 I am grateful to an anonymous reviewer for this journal for raising this issue.

11 See Beebe and Shea (2013) for attributions.

12 I realize that using the words consensus and orthodoxy may sound biased, and may come across as stylistically suboptimal. I believe I follow what is fairly standard in experimental philosophy at the moment, but hasten to say that my use of the term should not be taken to mean that I subscribe to the consensus/orthodoxy.

13 Bonferrni correction was applied in the following way. There are 7 SES classes, so each round involves $\frac{7 \times 6}{2} =21$ pairwise comparisons. So we have 21 pairwise comparisons for Watch, 21 for Banknote, and so on, and moreover 21 for all vignettes together, 21 for the Gettier vignettes together, and so on, that is, 12 of such rounds, or a total of 12×21 = 252 pairwise comparisons. Bonferroni correction is for each of the 12 rounds, which yields corrected *p* values by multiplying unadjusted *p* values by 21. Also note that these findings are not substantially changed were we to adopt the less conservative Benjamini–Hochberg procedure, or even if we looked at unadjusted comparisons. I report findings for unadjusted comparisons here for completeness. (Knowledge ascription throughout this footnote.) In Book (knowledge), Elite (74%) vs. Traditional Working Class (92%) and Precariat (91%), both* p* < .05. In Car (Gettier), Precariat (67%) vs. Established Middle Class (86%,* p* < .05), Technical Middle Class (92%,* p* < .05), New Affluent Workers (82%,* p* < .05), Traditional Working Class (89%,* p* < .01) and Emergent Service Workers (92%,* p* < .01). In Trip (Gettier), New Affluent Workers (19%) vs. Elite (0%,* p* < .05) and Established Middle Class (3%,* p* < .05). And in all Gettier cases together, Emergent Service Workers (24%) vs. New Affluent Workers (36%) and Precariat (40%), both* p* < .05.

14 Even assuming a small effect size (*w* = .1) leaves high power (.98).

15 Assuming *w* = .1 gives a power of .88.

16 There are 7 educational classes, which means that there are 21 pairwise comparisons for each round. I apply Bonferroni to each round; a corrected *p* value for a given pair in a given round is 21 times the uncorrected *p* value for that pair in that round.

17 An argument along these lines would have less force against using pairwise comparisons to test SES effects, at least if one uses the new SES measure, because the GBCS does not entail a straightforward ranking of SES groups. (This is incidentally why I used a great diversity of variables to capture SES (Elite, EMC, HighSES, etc.), even if Savage and colleagues do present one ordering, which I use in the final model with SES.).

18 Thanks to an anonymous reviewer of this journal for this reference.

19 The University of Groningen works with Joe Miele of MTurk Services, whose assistance is gratefully acknowledged here.

20 See https://www.bbc.com/news/magazine-34766169 [perma.cc/55EZ-23GY].

21 New Affluent Workers (20%) vs. Elite (80%,* p* < .05), Established Middle Class (67%,* p* < .05), and Precariat (71%,* p* < .01).

22 Technical Middle Class (38%) vs. Emergent Service Workers (67%) and Precariat (71%), both* p* < .01.
