# Supplementary material for: Knowledge attribution, socioeconomic status, and education: new results using the Great British Class Survey
Source: Synthese. 2021 Sep 12;199(3-4):7615–57. doi: 10.1007/s11229-021-03131-6 (PMC8435110; doi:10.1007/s11229-021-03131-6)
Supplement: Supplementary file 1 — Supplementary file1 (PDF 490 kb) [file 11229_2021_3131_MOESM1_ESM.pdf]

Supplementary Materials for: "Knowledge Attribution, Socioeconomic Status, and Education: New Results Using the Great British Class Survey," *Synthese*, accepted for publication 16 November 2020*

Boudewijn de Bruin†

3 February 2021

Abstract: This paper presents new evidence on the impact of socioeconomic status (SES) and education on knowledge attribution. I examine a variety of cases, including vignettes where agents have been Gettiered, have false beliefs, and possess knowledge (according to orthodoxy). Early work investigated whether SES might be associated with knowledge attribution (Weinberg, Nichols, and Stich 2001; Seyedsayamdost 2014). But these studies used college education as a dummy variable for SES. I use the recently developed Great British Class Survey (Savage et al. 2013) to measure SES. The paper reports evidence against an association between SES and patterns of knowledge ascription, and reports mixed evidence about education effects.

Keywords: Gettier, knowledge attribution/ascription, socioeconomic status (SES), Great British Class Survey, Latent Class Analysis, education

*This work is part of research project 360-20-310 financed by the Dutch Research Council (NWO).

†University of Groningen, Oude Boteringestraat 52, 9712 GL (philosophy), Nettelbosje 2, 9747 AE (economics), Groningen, The Netherlands, +31 (0)50 3633685, `b.p.de.bruin@rug.nl`, `www.bpdebruin.com`.

1 Introduction

This documents provides further information concerning the data, variable construction, comprehension questions, attention check, and replication of published results, as well as graphs for Study 3. Data are available online.

2 Study 3

I start describing variable construction for Study 3, the pilot. This is because variable construction is slightly more complicated there, and contains all elements necessary for variable construction in Study 1 (UK sample) and Study 2 (US sample). This section also contains graphs for Study 2.

2.1 SES variables

I derived posterior class assignments for all participants in the sample in the following way. I started with the Stata files used by Savage, which are contained in `gfk_cleaned_eul.dta` and `gbcs_cleaned_eul.dta`, respectively. Variables in these files were suffixed `_gfk` and `_gbcs`, respectively, for clarity. I merged these two files with the Stata file into one file, the result of which is called the "combined dataset." Henceforth I use *Savage* to refer to the methodology used by Savage's team.

Savage uses Latent Class Analysis (LCA) on the following six variables, of which I detail construction below:

- `zses_inc` standardized version of income
- `zses_assets` standardized version of assets
- `zses_occstatus` standardized version of mean status of occupations in social network
- `zses_occnumber` standardized version of number of occupations in social network
- `zses_acthigh` standardized version of high culture score
- `zses_actemer` standardized version of emerging culture score

zses_inc Savage has different categories to measure annual income after taxes. I collapsed Savage hhincome_gfk and hhincome_gbcs on income (suffixes omitted hereafter). Savage uses midpoints of the income, property, and savings categories, so I introduced a variable incomemid to capture the midpoints of income for the combined sample.

Savage standardizes all six variables using the means and standard deviations of the underlying variables restricted to the GFK sample, which is Savage's UK representative sample (the other, larger GBCS sample is obtained through the BBC website and suffers from selection bias). Since my income variable is different from the one Savage uses (because of the different categories), I calculated mean and standard deviation of the income variable restricted to the GFK sample, and used these to standardize incomemid for the combined dataset (M = 33572.42, SD = 27532.45). Pearson correlation of incomemid and hhincome_gfk is .894, $p < .001$, and for hhincome_gbcs .860, $p < .001$.

Note that for Study 1 and 2, it is not necessary to estimate the correlation between my measure and the Savage measure, because I used the very same items as they did.

I asked respondents: "What is your annual household income after taxes?" Answer brackets: under £10,000, £10,000–£25,000, £25,000–£50,000, £50,000–£100,000, over £100,000.

zses_assets Assets capture the sum of property and savings. I adopted less fine-grained categories than Savage to measure property and savings, so I recoded and introduced propertymid and savingsmid in the combined dataset to capture the midpoints of these variables. Pearson correlations between propertymid and housemid_gfk (the relevant variable in Savage) is .913, $p < .001$, and housemid_gbcs is .463, $p < .001$; and between savingsmid and savmid_gfk is .948, $p < .001$, and savmid_gbcs is .962, $p < .001$.

I follow Savage in deriving a measure of assets from these. Savage standardizes the property and savings variables first (with M = 145307.7, SD = 120476.3 for property, and M = 19726.69, SD = 39617.3 for savings), producing zses_prop and zses_sav, and then sums and standardizes (M = −.0731745, SD = 1.650294) to obtain zses_assets. I find this less statistically appealing than summing the midpoint variables first, and then standardizing (M = 155170, SD = 135609.2). As our results go through on both measures, I follow Savage here, though.

In the original Savage dataset there is no variable for assets (unlike variables for income, property, and savings), so I cannot directly compute Pearson correlations

to estimate how close the approximation is. So I defined a variable assets_old as the sum of savmid and housemid as per Savage's technical notes. Pearson correlations are .910, $p < .001$ between this variable and zses_assets on the GFK sample, and .733, $p < .001$, on the GBCS sample.

I asked respondents: "What is the value of all property owned/mortgaged by you/spouse/significant other?" Answer brackets: up to £125,000, £125,000–£250,000, £250,000–£500,000, over £500,000. And I asked respondents: "Do you have any savings (pensions, shares, ISAs, etc.)?" Answer brackets: none (coded £0), up to £10,000, £10,000–£25,000, £25,000–£50,000, £50,000–£100,000, over £100,000.

zses_occstatus In Savage, skmean is the mean CAMSIS (Cambridge Social Interaction and Stratification Scale) score of a respondent's social contacts. I included questions about a subset of the occupations from the Savage survey (secretary, nurse, teacher, cleaner, university lecturer, artist, electrician, office manager, solicitor, farm worker, chief executive, software designer, call centre worker, postal worker, scientist, lorry driver, accountant, shop assistant). This selection coincides with the questions currently online at the BBC website perma.cc/9YDT-M26W. I mapped the relevant responses in the two Savage samples to occ_secretary, occ_nurse, etc. I then calculated occstatus as the weighted average of these 18 items, with weights as in Savage (secretary = 47.49, nurse = 36.78, teacher = 76.46, cleaner = 10.73, university lecturer = 85.3, artist = 42.95, electrician = 24.44, office manager = 59.3, solicitor = 75.11, farm worker = 14.47, chief executive = 70.82, software designer = 51.48, call centre worker = 27.96, postal worker = 23.06, scientist = 68.77, lorry driver = 14.5, accountant = 57.51, shop assistant = 31.32). A respondent gets, say, a score of $(47.49 + 36.78)/18$ if they know exactly a secretary and nurse socially. I standardized occstatus for the combined dataset (M = 19.29248, SD = 11.08383), producing zses_occstatus.

zses_occnumber In Savage, sknum counts the number of different occupations the respondent has indicated are part of their network. In the above example, it would be 2. As I use a subset of occupations, I introduced a new variable occnumber, and standardized (M = 7.849903, SD = 4.092931).

zses_acthigh Savage introduces chigh as "simply 2 less than sum [*sic*] of the 9 variables that were most distinctive in the MCA [multiple correspondence analysis] [described in (Savage et al. 2013)] these are: ffrench, a transformed

version of eatfren, and carts, cmusgall, cstathom, ctheatre, copera, mclassic, mjazz, ffrench, cdance" ("Methodology and Technical Notes," p. 13). This does not seem entirely correct, as mclassic and mjazz have reversed coding. I corrected this. Moreover, my sample does not contain ffrench (which captures the respondent's preference for French food).

I therefore developed a variable that is as close as possible to chigh, namely, acthigh, which just as occnumber counts the number of activities a respondent is engaged in, from the following collection: doing arts and crafts, going to museums, going to stately homes, going to the theatre, going to the opera, listening to classical music, listening to jazz, and watching dance or ballet. This is a slightly cruder measure than Savage uses, but still with good correlation with chigh (.886, $p < .0001$, for the GFK sample, and .794, $p < .0001$ for the GBCS sample).

In my sample, variables artscraft, act_museums, etc. capture answers to "yes/no" questions. Savage offers a four-point Likert scale (0 = "never," 1 = "rarely," 2 = "sometimes," 3 = "often") for engagement in activities, and a five-point Likert scale for preferences for (some) types of music (1 = "like a lot," 2 = "like," 3 = "neither like nor dislike," 4 = "dislike," 5 = "dislike a lot"). I recoded, where 0 and 1 were taken to indicate "no," and 2 and 3 as "yes" (for the activities); and 0, 1, and 2 were taken to indicate "no," and 3 and 4 "yes" (for the music preferences).

Having introduced acthigh as described above, I define zses_acthigh as the standardized version (*M* = 1.88499, *SD* = 1.803636).

zses_actemer Savage writes that "cemer is the emerging cultural capital measure used in the analysis. It is 2 less than the sum of the 10 variables that were most distinctive on that dimension in the MCA [multiple correspondence analysis, seen main text of article]: ccompgam, csocnet, csportp, csportw, cfriends, cgym, cgig, mrap, cnet, mrock" ("Methodology and Technical Notes," p. 13). We get as close to this by defining a variable actemer analogously to acthigh, as the sum of the following activities: playing video games, using Facebook/Twitter, watching sports, socializing at home, going to the gym, going to gigs, listening to hiphop, listening to rock/indie. I leave out the activity of doing sports (csportsp) and surfing internet (cnet), as I did not include these items in the survey. actemer and cemer have high correlation on the GFK sample (.845, $p < .0001$), which is the main criterion of success because the GFK sample is the Savage sample that is representative of the UK population. There is lower correlation on the GBCS sample, which is the non-representative 300,000 plus sample generated

through the BBC website (.552, p < .0001). I standardized to `zses_actemer` (M = 3.311891, SD = 1.841578).

2.2 SES data

Savage used two samples. One is the GFK sample, contained in `gfk_cleaned_eul.dta`. It has 1,026 observations, and is based on research conducted by GfK, a UK polling firm, in April 2011. While as close to being representative of the UK population as is practically feasible, Savage slightly reweighted observations with weights captured in `weight_gfk`. The GBCS sample was gained from Savage cooperation with BBC, who ran the survey through a website from 2011 to 2013 (wave 1 from January to June 2011, with 161,399 observations, wave 2 from July 2011 to July 2013, with 164,313 observations), contained in `gbcs_cleaned_eul.dta`. This GBCS sample (the BBC sample) is not representative as it suffers from selection bias. That is why Savage assigns weights: observations from wave 1 of GBCS receive weight 1/161400, and observations from wave 2 of GBCS receive weight 1/1000000000.

The sample is derived from an opt-in internet panel. Such panels do not generally provide nationally representative results (Yeager et al. 2011). This applies to my sample as well, which is disproportionally unreligious, white, well-educated, and affluent, and includes disproportionally many participants aged 25 to 45 years. This may hamper the adequate assignment of posterior class membership to individual participants. Reweighting is seen as a partial remedy. I used UK census data (2011) to reweight the results (only for the purposes of assigning a measure of socioeconomic status, so all later analyses are carried out unweighted samples), using the entropy weighting procedure that is gaining traction in the literature (Hainmueller and Xu 2013). Weights are captured in `weight`, and I expanded this variable to include the Savage weights of the GFK and GBSC samples too.

Variables `cluwt_gfk` and `NEWcluwt_gbcs` capture the class assigned by Savage's LCA to any respondent. It takes seven values: 1 = "Elite," 2 = "Established Middle Class," 3 = "New Affluent Workers," 4 = "Technical Middle Class," 5 = "Traditional Working Class," 6 = "Emergent Service Workers," 7 = "Precariat." I introduced a new variable `knownclass` copying these variables.

I used the Latent Gold software package (version 5.1.0), which is the software that Savage uses. In all analyses, the `knownclass` variable was used to ensure that class assignment of respondents from the samples from Savage remained unchanged. I ran as per the research design of Savage by regressing a

latent class outcome variable (with 7 classes pre-assigned) on the six predictor variables `zses_income`, `zses_assets`, `zses_occstatus`, `zses_occnumber`, `zses_actemer`, and `zses_acthigh`. The resulting variable is `class_savage`. I did not attempt to determine better models on the basis of information criteria, as I worked on the assumption that Savage has shown seven classes as optimal in a UK context. Akaike's Information Criterion (AIC) was 34288.5, Bayesian Information Criterion (BIC) was 34820.8, for the combined reweighted sample of 2737.0 observations (weighted number).

Eight variables were derived from the LCA: seven variables `clu_`*n* capture the probability of a respondent being a member of class *n*. `clu_` takes *n* for the class *n* that has the highest probability (that is, for which `clu_`*n* is highest). I define an ordinal measure of SES `ses`, following the standard ordering as per Savage.

2.3 Knowledge attribution data

Data were gathered in the context of a larger experiment on the effects of learning and other exogenous factors on knowledge attribution (1,710 participants, 826 female, mean age 41 years). I use only the baseline sample from this experiment (in which participants were randomly assigned to the Watch, Banknote, or Book vignette), which is called *Sample A*, with 436 observations and a subset of the observations from the learning sample (which includes Car, Match, Trip, and Politician), which is called *Sample B*, with 343 observations.

This design led to the following exclusion criteria, which were made prior to the experiment and analysis.

First, I did not include comprehension questions. In a study on the impact of learning and other environmental factors on knowledge attribution, asking comprehension questions would risk substantially conflating results, and hence I did not present comprehension questions to any of the 1,710 participants. A result of this is that we may find knowledge attribution rates that are different from what researchers have found in studies that do use comprehension questions. This applies to the entire sample for Study 3. I was originally not too concerned about this issue, but thoughtful comments of an anonymous reviewer convinced me that more reliable results should be obtained by including comprehension questions. That is why I ran a new study with the same vignettes, with comprehension questions (that is, Study 1), and why I also ran the US study with comprehension questions (Study 2).

Secondly, the participants in Sample B were confronted with a battery of vignettes on some of which they received feedback, as I wanted to examine the

effects of sequential learning and feedback on knowledge attribution. This feedback concerned such things as whether their answer was in line with philosophical orthodoxy. So I decided to use from Sample B only the answer to the first vignette that a participant was confronted with. As a result, for all participants and observations that I use in the pilot (so for Sample A and B) the vignette we consider is the first they saw, was preceded by fully general instructions, and was not preceded or followed by comprehension questions. Subjects were unable to return to earlier stages of the survey. In Studies 1 and 2 reported in the paper and discussed later in the Supplementary Materials, I do not confront participants with more than one vignette.

Thirdly, to keep the survey of Study 3, the pilot, to a manageable size (note that I included further items for learning and other environmental factors), I only used a subset of the questions from the Great British Class Survey, namely, those that are included in the online version maintained by the BBC for the general public, at `perma.cc/9YDT-M26W`. In Studies 1 and 2, by contrast , I use the full range of questions that Savage uses.

A fourth element that has to be stressed is that I did not conduct an a priori power analysis specifically targeted to the purposes of the pilot. For Studies 1 and 2, I did run a prior power analysis.

Let me give more detail on the two samples. Sample A contains 436 observations (216 female, 38 years) involving one of three cases (Watch, Banknote, Book) drawn from Starmans and Friedman (2012), Experiment 1A (387 after removing duplicates, etc., 199 female, mean age 38). To adjust to the UK context, "dollar bill" was changed to "banknote." Sample B includes 343 observations (169 female, 38 years) in which subjects were exposed, in random order, to four cases (Car, Match, Trip, and Politician), totaling 322 after removing duplicates, etc. (162 female, mean age 38).

There are two reasons why I aggregate A and B for some of the analyses. The first is straightforward: it allows us to study the effects of SES on Gettier, false belief, and knowledge vignettes and other categories mentioned in the main text of the article. A second reason to combine A and B is that it allows us to estimate more accurate posterior probabilities of SES group membership. In fact, I conduct the LCA on an even larger sample containing the original 1,710 observations (which includes A and B, plus further unrelated observations concerning learning and other environmental factors) plus the samples used by Savage, for which posterior class probabilities are already available. It is on this grand set of more than 325,000 observations that I conduct LCA. We can do this because I asked all of the 1,710 participants to answer the GBCS questions.

Some information about the sample used by Savage is in order. This is important also for the UK study reported in the paper, as I use the same technique there (that is, I combine my sample with theirs). Savage draws from two surveys. One contains 325,712 observations (161,399 in a first wave, 164,313 in a second wave). Since respondents were approached through BBC marketing, this part of the sample suffers some selection bias. A survey of 1,026 observations conducted by a professional polling firm, GfK, is the second survey. To estimate class membership by means of LCA, Savage weights observations from the two waves of the BBC sample (first wave: 1/164400; second wave: 1/1000000000). Since also the sample from the second survey (by GfK) is not fully nationally representative, they give weights to that second survey in line with the most recent UK census. The sample of 1,710 observations is similarly reweighted for the purposes of LCA. I use Hainmüller and Xu's (2013) entropy balancing procedure.

2.4 Graphs

The main paper did not contains graphs for Study 3. I here illustrate weighted knowledge ascription per vignette (Figure 1), knowledge ascription per vignette (Figure 2), and knowledge and consensus ascription per SES group (Figure 3). I also include two graphs plotting SES membership and consensus ascription in three-dimensional space (economic, social, cultural capital, Figure 4).

Comparing these graphs with their analogs in Study 1 helps appreciating the differences between the underlying samples, in particular, the fact that in Study 3 no comprehension checks were used. Tentatively, one should think that the data for TMC (Technical Middle Class) appear to be outliers.

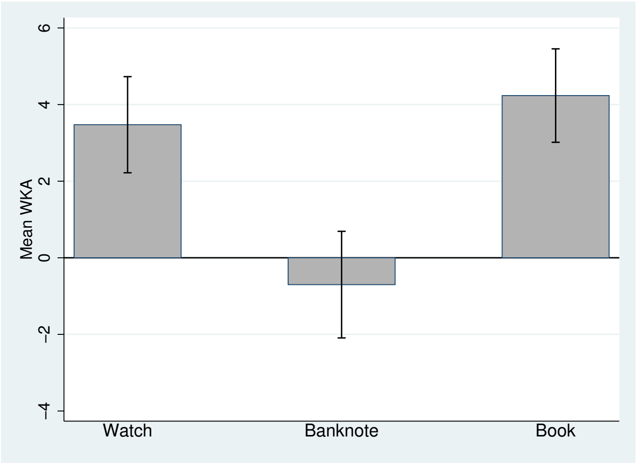

Figure 1: Weighted Knowledge Ascription per Vignette (Study 3)

n = 436. The figure displays the mean weighted knowledge ascription (WKA) per vignette, which was significantly different from chance in all vignettes (see main text). Scale is from −4 to 6. Error bars indicate 95% confidence intervals. Watch is a Gettier case. Banknote is a false belief case. Book is a knowledge case. No confidence ratings were obtained for Car, Match, Trip, and Politician in Study 3.

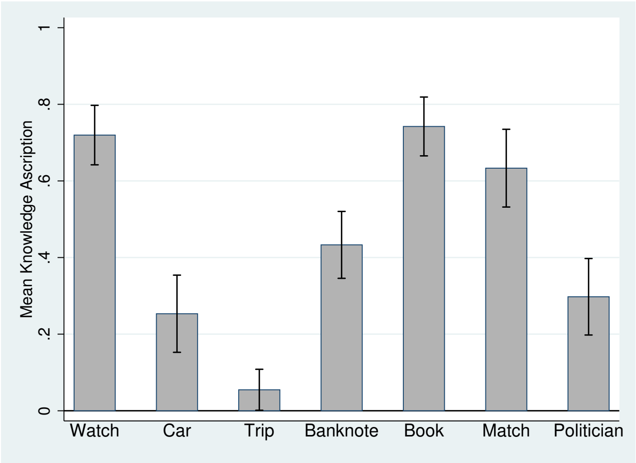

Figure 2: Knowledge Ascription per Vignette (Study 3)

n = 709. The figure displays the mean knowledge ascription per vignette, which was significantly different from chance in all vignettes (see main text). Error bars indicate 95% confidence intervals. Watch, Car, and Trip are Gettier cases. Banknote is a false belief case. Book, Match, and Politician are knowledge cases.

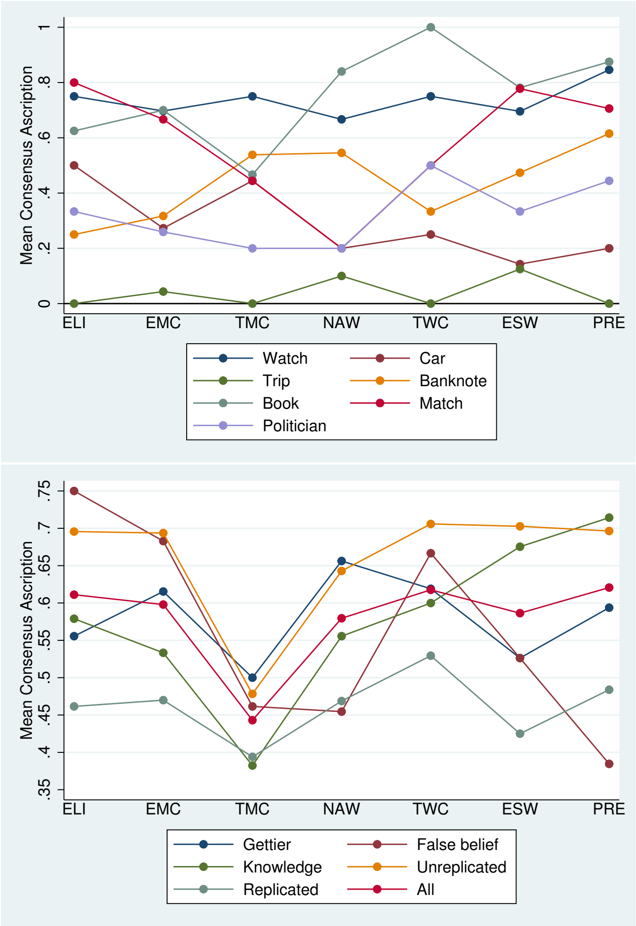

Figure 3: Knowledge Ascription and SES per Vignette, and per Group of Vignettes (Study 3)

The graphs in the upper panel plot the mean knowledge ascription for each of the seven SES groups, per vignette (n = 709). Watch, Car, and Trip are Gettier cases. Banknote is a false belief case. Book, Match, and Politician are knowledge cases. The graphs in the lower panel plot the mean consensus ascription for each of the seven SES groups, for various groups of vignettes. Consensus ascription is 1 if the knowledge/belief question was answered in line with epistemological consensus, and 0 otherwise. Scale is from .35 to .75. Gettier (n = 629) includes Watch, Car, and Trip. False belief (n = 224) includes Banknote. Knowledge (n = 811) includes Book, Match, and Politician. Unreplicated (n = 647) includes Banknote, Trip, and Politician. Replicated (n = 1017) includes Watch, Book, Car, and Match. All (n = 1664) includes all seven vignettes. ELI = "Elite," EMC = "Established Middle Class," TMC = "Technical Middle Class," NAW = "New Affluent Workers," TWC = "Traditional Working Class," ESW = "Emergent Service Workers," and PRE = "Precariat." The order of the seven groups follows Savage et al. (2013).

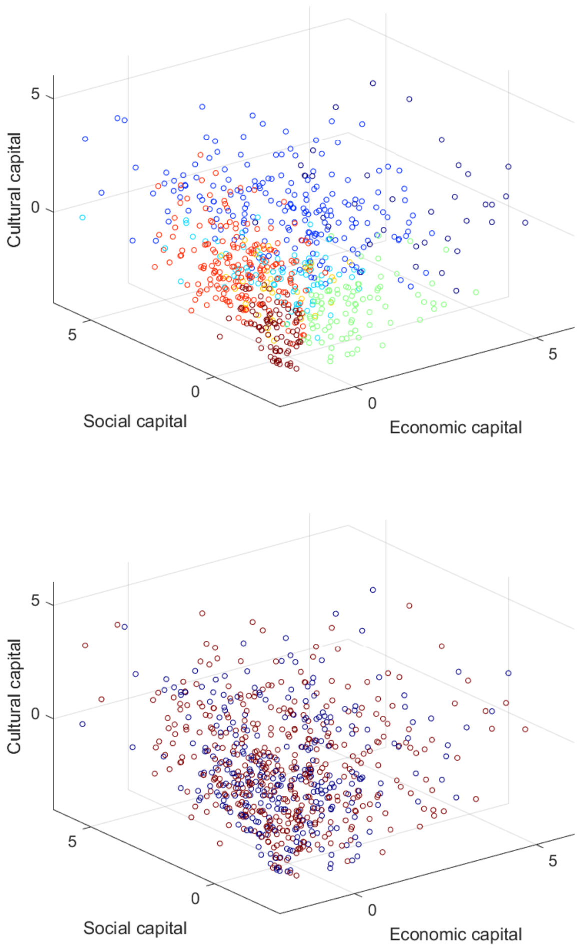

Figure 4: Economic, Social, and Cultural Capital and SES Group Membership (upper panel), and Consensus Ascription (lower panel) (Study 3)

n = 709. The graphs plot each subject in three-dimensional space. Economic capital is measured by the sum of income and assets. Social capital is the sum of the mean status of one's social contacts and the number of one's social contacts. Cultural capital is the sum of the high and emerging culture scores. For the six underlying variables standardized versions are used. In the upper panel, the color of each subject represents the SES group to which they are assigned using Latent Class Analysis as per Savage et al. (2013). Orange = "Elite," yellow = "Established Middle Class," green = "Technical Middle Class," blue = "New Affluent Workers," indigo = "Traditional Working Class," violet = "Emergent Service Workers," and red = "Precariat." In the lower panel, the color of each subject represents whether they answered the knowledge/belief question according to epistemological consensus (red) or not (blue).

3 Study 1

In line with the above variable construction for Study 3, and following Savage, I introduced six variables. The first two variables capture economic capital. There were three items: one for income, two for assets, so assets is the sum of a respondent's house value and remaining savings. I used brackets adjusted to the most recent UK census. I used midpoints for each bracket, and standardized the resulting variables. For income, participants were asked: "What is your annual household income after taxes?" Answer brackets: under £5,000, £5,000–£10,000, £10,000–£15,000, etc. until £100,000, and £100,000–£149,999, £150,000–£199,999, and over £200,000. For house value, they were asked: "What type of property do you live in?" Answer brackets: a rented property (coded £0), a property that you own worth under £50,000, a property that you own worth £50,000–£99,999, a property that you own worth £100,000–£149,999, etc., with last bracket: a property that you own worth over £500,000; and a final answer option: other (coded £0). For remaining assets, participants were asked: "What is the current value of your savings aside from your house?" Answer brackets: up to £999, £1,000–£1,999, £2,000–£2,999, etc., £20,000–£24,999, £25,000–£29,999, £100,000–£109,999, £110,000–£119,999, etc., with last bracket: over £200,000.

For social capital, I used the list of occupations from the full GBCS study, so not the restricted list that I used in Study 3, which was the BBC website list, but the list Savage uses. Participants were asked: "Think about your family, friends and acquaintances. What do they do? Here is a list of occupations. For each one, select it if you know someone socially who does that." The list was: secretary, travel agent, call centre worker, solicitor, accountant, scientist/researcher, clerical officer in national or local government, office manager, chief executive, bus or coach driver, finance manager, bank or building society manager, publican, restaurant manager, factory worker, receptionist, sales/shop assistant, university/college lecturer, civil/mechanical engineer, medical practitioner, electrician, gardener, train driver, nurse, teacher (primary/secondary school), software designer, sales or shop assistant, farm worker, security guard, postal worker, machine operator, catering assistant, artist/musician/performer, soldier, never worked, full-time student, aristocrat/nobility, with weights as per Savage specifications, and then averaged. These scores were then standardized. The other social capital variable is the number of occupations one knows socially, also standardized.

For cultural capital, participants were asked about preferred activities, music styles, and restaurants. Participants were told: "You will now be asked to consider a number of activities, and asked to indicate how much you participate (never,

rarely, sometimes, often).” This was followed by the list: watching TV, playing computer games, reading books, reading magazines, surfing the internet (except social networks), participating in online social networks, going to rock/modern music gigs, going to classical music concerts, going to watch dance/ballet, going to the opera, playing sports, going to watch sports (live), going out to eat, entertaining guests in your home, going to a sports/leisure club, going to museums/galleries, gardening/walking outdoors, pub/bar/cafe/nightclub, bingo, stately homes/historic sites, theatre or musicals. Participants were also asked: “You will now be asked to consider a number of types of music, and asked to indicate how much you like them (like very much, like, neutral, dislike, dislike intensely).” This was followed by the following list: rock including indie, classical including opera, modern jazz, world, electronic including techno and house, urban including hiphop and rap, heavy metal, and country and western. Finally, they were asked: “If you were to choose a place to eat out, which would you like best, second best, and least?” This was followed by the following list: café or teashop, pizza restaurant, fast food/burger bar, fish and chips restaurant, pub/wine bar/hotel, Indian, Chinese/Thai, Italian, French, steakhouse, vegetarian restaurant, none of these.

For highbrow cultural capital, following Savage, I added the Likert scale score answers of respondents to the following activities: books, classical music, opera, museums, historic sites, theater and musicals; plus the inverse of a preference for classical music. This variable was then standardized.

For emerging cultural capital, and again following Savage, the same procedure was followed with respect to: computer games, internet, social networks, playing sports, watch sports, entertain guests, sports club, plus the inverse of prefernece for rock/indie and urban. This variable was then standardized.

To standardize, for all these variables the mean and standard deviation are taken of the sample of 1,706 observations, which is the entire sample minus unfinished, duplicate IP addresses, outlier completion times, and notably those that failed the stringent astrology/sports attention check (which is different from the comprehension checks), the text of which can be found below.

4 Study 2

I followed variable construction as per Savage. The first two variables capture economic capital. There were three items: one for income, two for assets: assets is the sum of a respondent’s house value, and remaining assets. I used brackets and terminology adjusted to 2018 US governmental data collection. I used midpoints

for each bracket, and standardized the resulting variables. For income, respondents were asked: "What is your annual household income after taxes?" Answer brackets: up to $10,000, $10,000–$14,999, $15,000–$24,999, $25,000–$34,999, $35,000–$49,999, $50,000–$74,999, $75,000–$99,999, $100,000–$149,999, $150,000–$199,999, $200,000 or above. For house value, respondents were asked: "What is the value of the home/property owned/mortgaged by you/spouse/significant other?" Answer brackets: I live in a rented home/property (coded $0), up to $50,000, $50,000–$99,999, $100,000–$149,999, $150,000–$199,999, $200,000–$299,999, $300,000–$499,999, $500,000–$999,999, $1,000,000 or above. For remaining assets, respondents were asked: "What is your net worth (excluding equity in your own home)?" Answer brackets: zero or negative (coded $0), $1–$4,999, $5,000–$9,999, $10,000–$24,999, $25,000–$49,999, $50,000–$99,999, $100,000–$249,999, $250,000–$449,999, $500,000 or above.

Education was measured using a categorical variable in line with US statistical conventions (1 = "Less than 9th grade," 2 = "9th to 12th grade, no diploma," 3 = "High school graduate," 4 = "Some college (but no degree), or associate's degree or equivalent level," 5 = "Bachelor's degree or equivalent level," 6 = "Master's degree or equivalent level," 7 = "Doctoral degree or equivalent level").

For social capital, I used the list of occupations that Lin (2001), the researcher behind the position generator approach that Savage uses, has developed specifically for the US. It includes exactly the following items: elementary school teacher, lawyer, salesperson, waiter/waitress or bartender, engineer, secretary, manager, small business owner, insurance agent, janitor, mechanic or repairman, laborer, foreman, and skilled worker. Lin also provides us with the weights (social status) that we should attach to these occupation. I report these weights here: schoolteacher = 64, lawyer = 75, salesperson = 32, waiter = 28, engineer = 71, secretary = 46, manager = 51, business owner = 59, insurance agent = 45, janitor = 22, mechanic = 44, laborer = 24, foreman = 54, skilled worker = 30. Weighted averages are taken and then standardized. The questionnaire included a larger list of occupations, which might have been useful if Lin's position generator had failed to work here, for then I would have developed a new positional scheme: elementary school teacher, lawyer, salesperson, waiter/waitress or bartender, engineer, secretary, manager, small business owner, insurance agent, janitor, mechanic or repairman, laborer, foreman, skilled worker. These occupations were based on the GBCS list.

The other social capital variable is the number of occupations one knows socially. This variable is also standardized.

For highbrow cultural capital, I added the Likert scale score answers of respon-

dents to the following activities: books, classical music, opera, museums, historic sites, theater and musicals; plus the inverse of a preference for classical music. This variable is then standardized.

For emerging cultural capital, the same procedure is followed with respect to: computer games, internet, social networks, playing sports, watch sports, entertain guests, sports club, plus the inverse of prefernece for rock/indie and urban. This variable is then standardized.

Here too the list of items was larger than what I ultimately used. If the method used by Savage and in the Hungary (Albert et al. 2018) and Australia (Sheppard and Biddle 2017) studies, which I follow here, hadn't worked, I expected I would have had some chance of developing new variables with a larger set of items, using multiple correspondence analysis and/or other dimension reduction techniques. Fortunately that move was not necessary. Still I list all survey items here. Questions about cultural capital involved the following list of activities: watching TV, playing computer games, reading books, reading magazines, going to classical music concerts, surfing the internet (except social networks), participating in online social networks, going to rock/modern music concerts, going to watch dance/ballet, going to the opera, playing sports, going to watch sports (live), going out to eat, entertaining guests in your home, going to a sports/leisure club, going to museums/galleries, gardening/walking outdoors, pub/bar/cafe/nightclub, bingo, historic sites, theater or musicals, playing tennis, gourmet cooking, going to bluegrass concerts, barbecuing, going bowling. Participants were also asked about music preferences from the following list: rock including indie, classical including opera, jazz, world, electronic including techno and house, urban including hiphop and rap, heavy metal, country and western, pop, R&B and soul, blues, reggae, Christian or gospel. Finally, they were asked about their preference for restaurants from the following list: café or teashop, pizza restaurant, fast food/burger bar, pub/wine bar/hotel, steakhouse, vegetarian, American, Italian, Mexican, Chinese, Japanese/sushi, Thai, French, Indian, none of these. This choice of items was based on my reading of some relevant literature on class in the US as well as on Pew data on music and restaurant preferences in the US.

To standardize, for all these variables the mean and standard deviation are taken of the sample of 974 observations, following the same exclusion criteria as in Study 1.

Weights were assigned using entropy balancing (Hainmueller and Xu 2013) to boost national representativeness with data drawn from the most recent publicly available 2018 and 2019 Unites States Census Bureau data, except for religion (data about which the US government is not allowed to collect), drawn from the

Number of classes	LL	AIC	BIC
6	−6310.95	12775.91	13151.77
7	−6144.76	12469.52	12908.85
8	−5896.51	11999.02	12501.80
9	−5799.82	11831.64	12397.88
10	−5710.72	11679.44	12309.14
11	−5603.09	11490.18	12183.34
12	−5432.98	11175.96	11932.58
13	−5467.26	11270.51	12090.59

Table 1: Latent Class Analysis

Note. The first column indicates the number of classes in the model. The next three columns give the corresponding log-likelihoods (LL), Akaike information criterion (AIC), and Bayesian information criterion (BIC).

Pew Research Center Religious Landscape Study 2019. Ultimately, balancing did not make a difference to our results, and that is why I report unbalanced results only.

As Table 1 shows, the best fit is a model of 12 classes. But just as the original UK study, the Hungary study, and the Australia study, I opt for a lower number of classes because models will otherwise have very small classes. With all models with more than seven classes, we would have classes of less than a hundred members, often much smaller than that. I therefore take the seven-class model as my point of analysis, in line with the UK, Hungary, and Australia studies.

The Latent Gold software package (version 5.1.0), which Savage also deploys, was used to produce posterior probability estimates of class membership, explained in the article. These, plus the final class assignment, are the basis of the analysis.

5 Comprehension checks

Starmans and Friedman (2012) use comprehension check questions to test whether participants have read and understood the vignettes. I included the very same questions for the three vignettes drawn from their paper (Watch, Banknote, Book), and used the same presentation conventions (the text of the vignette is shown while the question plus answer options appear). For the four vignettes drawn from other sources (Car, Match, Trip, Politician) I developed comprehension checks

with the aim of making them as analogous to the three sets of questions developed by Starmans and Friedman as possible. I list the comprehension check questions here below, together with the texts of the vignettes.

Joe Miele of MTurk Data services, with whom the University of Groningen collaborates, brought to my attention that the wording of the second comprehension check question (in the Watch vignette, as per Starmans and Friedman (2012)) may be slightly ambiguous. It asks how the watch got on the table, but since the vignette features two watches that were on the table at some point in time (but not at the same time), respondents may interpret the question either as referring to the watch that is now on the table (which was placed there by a burglar), or to the watch that was on the table at the beginning of the vignette (and was placed there by Peter). That is why I use two ways to measure comprehension, one testing whether all four comprehension questions have been answered correctly (which is Starmans and Friedman's (2012) measure), the other whether the first, third, and fourth comprehension question have been answered correctly. The ambiguity does not hold for all of our seven cases, but the second question is always a supposedly factual question, so eliminating this question affects all seven vignettes equally. With this weaker test, my findings remain valid. I only report results that use the exact same exclusion criteria as Starmans and Friedman use, except for the results that we get for the entire sample, reported in the last subsection of the relevant section in the article.

Here follow the texts of the vignette and the comprehension questions (answer options between brackets).

Watch

> Peter is in his locked apartment reading, and is about to have a shower. He puts his book down on the coffee table, and takes off his black plastic watch and leaves it on the coffee table. Then he goes into the bathroom. As Peter's shower begins, a burglar silently breaks into the apartment. The burglar takes Peter's black plastic watch, replaces it with an identical black plastic watch, and then leaves. Peter is still in the shower, and did not hear anything.
>
> Is there a watch on the table? (Yes, No)
>
> How did the watch get on the table? (Peter put it there, The burglar put it there)
>
> Would Peter say there is a watch on the table? (Yes, No)

Why would Peter say there is a watch on the table? (Because Peter put a watch on the table, Because a burglar put a watch on the table)

Banknote

Peter is in his locked apartment reading, and is about to have a shower. He puts his book down on the coffee table, and takes off his black plastic watch and leaves it on the coffee table. Then he goes into the bathroom. As Peter's shower begins, a burglar silently breaks into the apartment. The burglar takes Peter's black plastic watch, replaces it with a banknote [in the US study: "dollar bill"], and then leaves. Peter is still in the shower, and did not hear anything.

Is there a watch on the table? (Yes, No)

How did the watch get on the table? (Peter put it there, The burglar put it there)

Would Peter say there is a watch on the table? (Yes, No)

Why would Peter say there is a watch on the table? (Because Peter put a watch on the table, Because a burglar put a watch on the table)

Book

Peter is in his locked apartment reading, and is about to have a shower. He puts his book down on the coffee table, and takes off his black plastic watch and leaves it on the coffee table. Then he goes into the bathroom. As Peter's shower begins, a burglar silently breaks into the apartment. The burglar takes Peter's black plastic watch, replaces it with an identical black plastic watch, and then leaves. Peter is still in the shower, and did not hear anything.

Is there a book on the table? (Yes, No)

How did the book get on the table? (Peter put it there, The burglar put it there)

Would Peter say there is a book on the table? (Yes, No)

Why would Peter say there is a book on the table? (Because Peter put a book the table, Because a burglar put a book on the table)

Car

Bob has a friend, Jill, who has driven a Buick for many years. Bob therefore thinks that Jill drives an American car. He is not aware, however, that her Buick has recently been stolen, and he is also not aware that Jill has replaced it with a Pontiac, which is a different kind of American car.

Does Jill drive an American car? (Yes, No)

What car does Jill drive? (Jill drives a Pontiac, Jill drives a Buick)

Would Bob say Jill drives an American car? (Yes, No)

Why would Bob say Jill drives an American car? (Because Jill has driven a Buick for many years, Because Jill has driven a Pontiac for many years)

Match

A pyromaniac has just purchased a box of Sure-Fire Matches. He has done so many times before and has noted that they have always lit when struck unless they were wet. Furthermore, he knows that oxygen must be present for things to burn and that the observed regularity between the matches' being struck and their lighting is not a mere coincidence. After perceiving that the matches are dry and that there is plenty of oxygen present, he proceeds to strike one of the matches, confident that it will light. It does.

Did the match light? (Yes, No)

Why did the match light? (The match was dry and there was plenty of oxygen, The match was wet and there was insufficient oxygen)

Would the pyromaniac say the match would light? (Yes, No)

Why would the pyromaniac say the match would light? (Because the matches were dry and there was plenty of oxygen, Because Sure-Fire Matches light when they are wet)

Trip

Luke works in an office with two other people, Victor and Monica. All winter Victor has been describing his plans to go to Las Vegas on

his vacation, even showing Luke the website of the hotel where he has reservations. When Victor is away on vacation, Luke sees Victor's Facebook photos of himself with Vegas landmarks in the background, together with status updates about how much he is enjoying his trip. When he gets back to work, Victor talks a lot to Luke about how much fun he had vacationing in Las Vegas. However, Victor didn't really go on the trip; he has just been pretending. His tickets and reservations were cancelled because his credit card was maxed out, and he secretly stayed home in Markham, very skillfully faking the Facebook pictures using Photoshop. Meanwhile, Monica just spent a weekend vacationing in Las Vegas, but kept this a secret from all her co-workers.

Did one of Luke's co-workers recently vacation in Las Vegas? (Yes, No)

How is it that one of Luke's co-workers recently vacationed in Las Vegas? (Monica spent a vacation in Las Vegas, and she is one of Luke's co-workers; Victor spent a vacation in Las Vegas, and he is one of Luke's co-workers)

Would Luke say that one of his co-workers recently vacationed in Las Vegas? (Yes, No)

Why would Luke say that one of his co-workers recently vacationed in Las Vegas? (Because Victor talked to him about a vacation in Las Vegas, Because Monica talked to him about a vacation in Las Vegas)

Politician

A political leader is assassinated. A reporter on the scene sends news of the assassination to her news agency so that the story can be included in the day's final edition of the paper. Jill buys a copy of that paper and reads the story of the assassination that was dictated by the reporter who witnessed the event.

Has the political leader been assassinated? (Yes, No)

Who assassinated the political leader? (Jill, Someone else)

Would Jill say that the political leader has been assassinated? (Yes, No)

> Why would Jill say that the political leader has been assassinated? (Because Jill bought a copy of the paper in which the assassination was mentioned, Because Jill was present at the scene)

6 Attention check

The following attention check was used Studies 1 and 2. The check is a standard and highly selective test that the economics lab of the University of Groningen requires when data are gathered through Amazon Mechanical Turk (in collaboration with Joe Miele of MTurk data services).

> The astrology has always had a big influence on people's life. Every day, people check their horoscope based on their day of birth. For some people, this is more important than others, but fact is that it still provides for a highly profitable market. Some people claim that the sign of your horoscope also influences how thoroughly you read texts. This question is designed to study this. Below, you can see the question about which astrological sign you are. Ignore this question, but indicate your favorite sport instead. This question will help us to better understand the influence of horoscope signs on behavioral decision-making.
>
> Please indicate your astrological sign (for example, Aquarius, Pisces, or Aries) [with empty space for text entry]

7 Replication of published studies

The results of Study 1 replicate earlier work on Watch, Book, Car, and Match, but not those of Banknote, Trip, and Politician. While I do not have a knock-down explanation of this, I think that providing further information on the differences between my data and the data from earlier studies may be useful in this regard.

7.1 Banknote

To begin with Banknote, we find that 28% of people assign knowledge, as opposed to 11% in Experiment 1A of Starmans and Friedman (2012). The presentation of the vignette was, however, entirely identical, except that instead of "dollar bill" I inserted "banknote," to adjust to the UK context. It is highly unlikely that this

change is responsible for the different knowledge attribution rates. Moreover, I used exactly the same exclusion criteria as Starmans and Friedman, and presented the vignette with the same comprehension questions, and in the same order, using the same convention of displaying the questions and answer options together with the text of the vignette (so no recall required).

There are three differences in terms of the underlying demographics that may be relevant. To begin with, I use a UK sample, while Starmans and Friedman use a US sample. Secondly, Starmans and Friedman's sample consists for 70% of participants who selected "Some College" or "Bachelor's Degree," whereas in my sample this is 47%. Thirdly, in Starmans and Friedman's sample, 19% of respondents indicate that they have taken more than one course in philosophy, as opposed to 16% of my sample. Besides this, there is a difference in sample size, with Starmans and Friedman's study (for the Banknote vignette) involving 46 observations, and here ranging from 397 (no exclusion criteria, Sample 1, Table 2), to 144 (most stringent exclusion criteria, Sample 8, Table 2). Figures 5 and 6 represent the differences between the samples visually.

7.2 Trip

Results in Trip were also different. In Trip we find more than 90% of the respondents assigning belief, rather than knowledge, as opposed to 61% in the US and online subsample (Machery et al. 2017). Again let us note the differences between the samples. Machery and co-authors collected data from 521 respondents (US, India, Japan, Brazil), who were each confronted with four vignettes. Their ultimate sample counts 245 observations. I take as reference here their subsample of 64 US respondents. The reason is that only their US participants used Amazon Mechanical Turk, the online platform, and all others were paper–pencil. My sample is online only. Besides the knowledge/belief question, Machery and colleagues asked questions about justification, and they left out of their analysis those respondents who found that there was justification lacking in all of the four cases. I did not ask questions about justification. So the sample used here may include some respondents who would have denied justification and would have been excluded had I used the exclusion criteria of the ealier study. Secondly, I only asked respondents to consider one vignette, and can exclude order effects. The earlier study, by contrast, involved four vignettes, so cannot exclude order effects for about 75% of respondents, per vignette. Moreover, I asked four comprehension questions prior to confronting participants with the knowledge/belief questions (as per Starmans and Friedman (2012)). One comprehension question

was asked in the reference study after each of the four vignettes. Finally, the study also included a further question (in addition to the knowledge/belief question), with answer options: "feels like (s)he knows," and "feels like (s)he knows but (s)he doesn't actually know". Answers to this second question differ from the answers to the dichotomous question, with 75% attributing belief as opposed to 61% (on the US sample of the published study). If we take this second question as the basis of comparison, then we witness only a very slight decrease in the the statistical significance of the difference with my results ($p < .01$).

The replication failure may be due to differences in nationality. Machery and colleagues find differences across nationalities, ranging from 32.5% (India subsample) to 64.4% (Japan). The difference may also be driven by my more intense use of comprehension checks that preceded the dichotomous question. Further demographic differences are that my sample has a more even male–female distribution (41% male in the original study). Machery and colleagues do not report age and other demographics. Their sample size is 64. My sample ranges from 386 (no exclusion criteria, Sample 1, Table 2), to 103 (most stringent exclusion criteria, Sample 8, Table 2). Figures 5 and 6 represent the differences between the samples visually.

7.3 Politician

There were significantly different results in Politician too. I used exactly the same vignette as Beebe and Shea (2013), but there are some differences between the samples. Theirs is a subsample of 60 of a sample of 189 undergraduate college students (US). The question respondents were confronted with was different from the one I asked, in that they were asked to indicate whether they agreed or not (presumably on a five-point anchored Likert scale, but design details do not seem to be reported in their paper) with the statement that "Jill knows that the political leader has been assassinated." Answers were then collapsed into two natural dichotomous (knowledge/belief) categories. Also unlike the current study, no comprehension questions seem to have been asked. Demographics are not given for the subsample beyond age and gender. The mean age of 31 years is about 10 years less than for the current sample. My sample size ranges from 399 (no exclusion criteria, Sample 1, Table 2), to 152 (most stringent exclusion criteria, Sample 8, Table 2). Their sample size is 60. Figures 5 and 6 represent the differences between the samples visually.

Another reason may be that a respondent's answer to the Politician question may be moderated by political convictions and/or by the extent of their trust in

	Literature	1	2	3	4	5	6	7	8
Watch	72	69	76	76	75	73	74	78	74
Banknote	11	31**	31**	30**	26*	26*	27*	28*	28*
Book	88	88	87	86	87	87	85	87	87
Car	14	15	15	15	16	16	16	16	17
Match	78	68	68	68	69	69	69	71	68
Trip	39	11***	10***	11***	9***	9***	9***	8***	8***
Politician	65	35***	35***	32***	34***	34***	31***	32***	34***
Observations		2824	2486	1706	2104	2023	1433	996	1664

Table 2: Replication and Subsamples (Study 1)

Note. The table gives knowledge ascription rates as percentages. Sample 1 contains all observations, without any exclusion criteria, with 2,824 observations for all seven cases. Sample 2 contains all observations minus unfinished, duplicate IP addresses, and completion times below 60 seconds and over 1,000 second (2,486 observations). Sample 3 is Sample 2 minus observations that failed the astrology/sports attention check (total 1,706). Sample 4 is Sample 2 minus observations that failed the weak comprehension test (2,104 observations). Sample 5 is Sample 2 minus observations that failed the strong comprehension test (2,023 observations). Sample 6 is Sample 2 minus observations that failed the astrology/sports attention check and observations that failed the strong comprehension check (1,433 observations). Sample 7 is Sample 6 minus observations that had prior exposure to philosophy and/or to similar experiments about knowledge and judgment (996 observations). Sample 8 uses Starmans and Friedman's (2012) exclusion criteria (unfinished, duplicates, comprehension checks, similar experiments), plus outlier completion times. Pairwise comparisons are always relative to the literature (two-sided), with *, **, and *** indicating significance at the 5%, 1% and 0.1% level, respectively. Watch, Car, and Trip are Gettier cases. Banknote is a false belief case. Book, Match, and Politician are knowledge cases.

journalists. We cannot test directly for this, as I included no measure of trust in the surveys. Regressions on political orientation and response to Covid-19 news (the latter of which may vaguely be associated with trust in media) shows no significant relations. Future research should look into this question more rigorously.

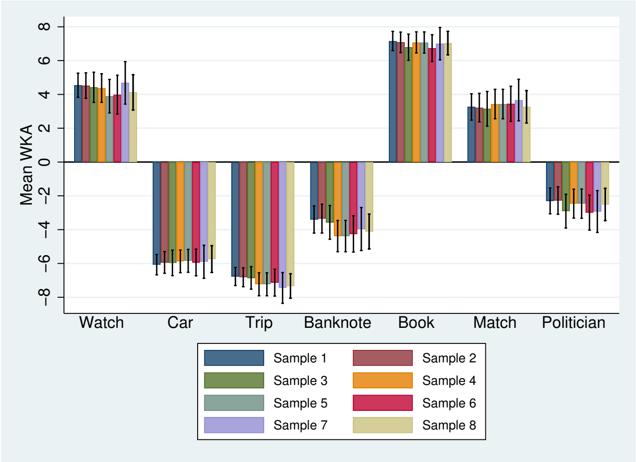

Figure 5: Weighted Knowledge Ascripion in Subsamples (Study 1)

The figure displays weighted knowledge ascription rates as percentages. Sample 1 contains all observations, without any exclusion criteria, with 2,824 observations for all seven cases. Sample 2 contains all observations minus unfinished, duplicate IP addresses, and completion times below 60 seconds and over 1,000 second (2,486 observations). Sample 3 is Sample 2 minus observations that failed the astrology/sports attention check (total 1,706). Sample 4 is Sample 2 minus observations that failed the weak comprehension test (2,104 observations). Sample 5 is Sample 2 minus observations that failed the strong comprehension test (2,023 observations). Sample 6 is Sample 2 minus observations that failed the astrology/sports attention check and observations that failed the strong comprehension check (1,433 observations). Sample 7 is Sample 6 minus observations that had prior exposure to philosophy and/or to similar experiments about knowledge and judgment (996 observations). Sample 8 uses Starmans and Friedman's (2012) exclusion criteria (unfinished, duplicates, comprehension checks, similar experiments), plus outlier completion times. Error bars indicate 95% confidence intervals. Watch, Car, and Trip are Gettier cases. Banknote is a false belief case. Book, Match, and Politician are knowledge cases.

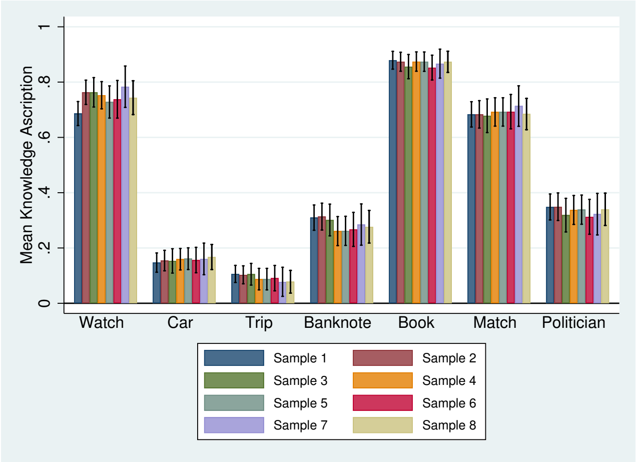

Figure 6: Knowledge Ascripion in Subsamples (Study 1)

The figure displays knowledge ascription rates as percentages. Sample 1 contains all observations, without any exclusion criteria, with 2,824 observations for all seven cases. Sample 2 contains all observations minus unfinished, duplicate IP addresses, and completion times below 60 seconds and over 1,000 second (2,486 observations). Sample 3 is Sample 2 minus observations that failed the astrology/sports attention check (total 1,706). Sample 4 is Sample 2 minus observations that failed the weak comprehension test (2,104 observations). Sample 5 is Sample 2 minus observations that failed the strong comprehension test (2,023 observations). Sample 6 is Sample 2 minus observations that failed the astrology/sports attention check and observations that failed the strong comprehension check (1,433 observations). Sample 7 is Sample 6 minus observations that had prior exposure to philosophy and/or to similar experiments about knowledge and judgment (996 observations). Sample 8 uses Starmans and Friedman's (2012) exclusion criteria (unfinished, duplicates, comprehension checks, similar experiments), plus outlier completion times. Error bars indicate 95% confidence intervals. Watch, Car, and Trip are Gettier cases. Banknote is a false belief case. Book, Match, and Politician are knowledge cases.

8 Covid-19 items

I included a number of questions about Covid-19, for a different project. They appeared after the knowledge attribution task and the demographic items, and will therefore not have influenced the results. I report them here for completeness.

The items were introduced by: "We will now ask you two questions about the coronavirus/COVID-19." Then I asked "Have you changed your daily routine in any way specifically because of the coronavirus/COVID-19? Select all that apply." Items were: "I have kept my children home from school," "I have worked from home," "I have canceled or altered upcoming travel plans," "I have recently purchased surgical masks or other items to shield me from the virus," "I have avoided physical contact with others, such as handshakes," "I am washing my hands, or using disinfectant more frequently," "I am avoiding large gatherings of people whenever possible," "I am avoiding public transportation," "Other," "I have not altered my daily routine," and "Don't know." Respondents were also asked: "Which of the following statements about the coronavirus/COVID-19 are true? Select all that you know are true." Items were: "The coronavirus cannot be transmitted in areas with hot and humid climates," "Cold weather and snow can kill the new coronavirus," "Taking a hot bath does not prevent the coronavirus disease," "The coronavirus can be transmitted through mosquito bites," "Hand dryers are effective in killing the coronavirus," "Ultraviolet disinfection lamps cannot kill the coronavirus," "Thermal scanners cannot detect people who are infected with the coronavirus but who are not yet sick with fever," "Spraying alcohol or chlorine all over your body kills the coronavirus," "Vaccines against pneumonia do not protect you against the coronavirus," "Regularly rinsing your nose with saline can help prevent infection with the coronavirus," "Eating garlic cannot help prevent infection with the coronavirus," "The coronavirus only affects older people," "Antibiotics are not effective in preventing and treating the coronavirus," "There are no specific medicines to prevent or treat the coronavirus yet," "Chlorine dioxide (aka MMS) can wipe out the coronavirus," "Colloidal silver has not been shown to treat or prevent the coronavirus," "Drinking water every 15 minutes flushes away the coronavirus," and "Drinking hot water and avoiding ice cream does not prevent or treat the coronavirus."

9 Political identity items

Study 1 (UK sample) also included the following question, towards the end, for a different project: "How did you vote in the 2019 General Election?" Options:

“Conservative,” “Labour,” “Liberal Democrats,” “Scottish National Party,” “Brexit Party,” “Green Party,” “Other,” “I didn’t vote,” “Don’t know,” and “Refuse.”

Study 2 (US sample) also included, towards the end: “With which political party do you most identify?” Options: “Strong Democrat,” “Moderate Democrat,” “Lean Democrat,” “Lean Republican,” “Moderate Republican,” “Strong Republican,” “Independent,” “Other,” “Don’t know,” and “Refuse.”

References

Albert, F., B. Dávid, Z. Kmetty, L. Kristóf, P. Róbert, and A. Szabó (2018). Mapping the post-communist class structure: Findings from a new multi-dimensional hungarian class survey. *East European Politics and Societies and Cultures 32*(3), 544–565.

Beebe, J. R. and J. Shea (2013). Gettierized Knobe effects. *Episteme 10*(3), 219–240.

Hainmueller, J. and Y. Xu (2013). ebalance: A stata package for entropy balancing. *Journal of Statistical Software 54*(7), 1–18.

Lin, N. (2001). *Social Capital*. Cambridge: Cambridge University Press.

Machery, E., S. Stich, D. Rose, A. Chatterjee, K. Karasawa, N. Struchiner, S. Sirker, N. Usui, and T. Hashimoto (2017). Gettier across cultures. *Noûs 51*(3), 645–664.

Savage, M., F. Devine, N. Cunningham, M. Taylor, Y. Li, J. Hjellbrekke, B. Le Roux, S. Friedman, and A. Miles (2013). A new model of social class? findings from the bbc’s great british class survey experiment. *Sociology 47*(2), 219–250.

Seyedsayamdost, H. (2014). On normativity and epistemic intuitions: Failure of replication. *Episteme 12*(1), 95–116.

Sheppard, J. and N. Biddle (2017). Class, capital, and identity in australian society. *Australian Journal of Political Science 52*(4), 500–516.

Starmans, C. and O. Friedman (2012). The folk conception of knowledge. *Cognition 124*(3), 272–83.

Weinberg, J. M., S. Nichols, and S. Stich (2001). Normativity and epistemic intuitions. *Philosophical Topics 29*(1-2), 429–460.

Yeager, D. S., J. A. Krosnick, L. Chang, H. S. Javitz, M. S. Levendusky, A. Simpser, and R. Wang (2011). Comparing the accuracy of rdd telephone surveys and internet surveys conducted with probability and non-probability samples. *Public Opinion Quarterly 75*(4), 709–747.
